# Optimal sizing and placement of hybrid PV-storage systems in microgrids using Bald Eagle Search Algorithm

**DOI:** 10.1038/s41598-026-56248-4

**Published:** 2026-06-11

**Authors:** Behnam Taherizadeh, Mehrdad Ahmadi Kamarposhti, Rahim Taherizadeh, Frank Werner, Phatiphat Thounthong, Ilhami Colak

**Affiliations:** 1 Department of Electrical Engineering, University College of Rouzbahan, Sari, Iran; 2 Department of Electrical Engineering, Jo.C., Islamic Azad University, Jouybar, Iran; 3 Department of Electrical Engineering, Imam Hussein University, Tehran, Iran; 4 Faculty of Mathematics, Otto-von-Guericke University, 39016 Magdeburg, Germany; 5 King Mongkut’s University of Technology North Bangkok, Renewable Energy Research Centre (RERC), 1518, Pracharat 1 Road, Wongsawang, Bangsue, Bangkok, 10800 Thailand; 6 Department of Electrical and Electronics Engineering, Faculty of Engineering and Applied Sciences, Istinye University, Istanbul, Turkey

**Keywords:** Bald eagle search algorithm, Battery storage, Photovoltaic systems, Power loss reduction, Voltage stability, Microgrid, Energy science and technology, Engineering

## Abstract

This study proposes an enhanced Bald Eagle Search (BES) optimization framework for optimal allocation and sizing of photovoltaic (PV) and battery energy storage systems (BESS) in active distribution networks. The objective is to minimize total operating cost and energy losses while improving voltage stability under deterministic load and generation conditions. The proposed approach is validated on IEEE 33-bus and IEEE 69-bus test systems under two scenarios: PV-only and integrated PV–BESS deployment. A comprehensive comparative assessment is conducted against Genetic Algorithm (GA) and Whale Optimization Algorithm (WOA) under identical operating conditions. The results demonstrate that the proposed BES algorithm consistently outperforms the benchmark methods. For the IEEE 69-bus system, BES achieves up to 41% and 55% reduction in daily energy losses for PV-only and PV–BESS cases, respectively, while improving the minimum bus voltage to 0.951 p.u. For the IEEE 33-bus system, BES yields 41.3% and 52% loss reductions under PV-only and PV–BESS configurations, respectively, and enhances the minimum voltage from 0.941 p.u. to 0.962 p.u. In both systems, BES also achieves the lowest operating cost among all compared methods. These findings confirm the robustness, scalability, and effectiveness of the proposed framework across different distribution network topologies.

## Introduction

In the past several decades, global energy use has climbed steadily, prompting various policies, goals, and strategies to mitigate carbon footprints, in light of concerns about pollution and greenhouse gas emissions from fossil fuels. The assimilation of renewable energy sources into the incorporation of various energy systems into support energy systems practices that corroborates a two-way flow of electricity between producers and customers showed substantial possibilities, especially for energy systems associated microgrids, or microgrid energy systems, which connect energy from intermittent renewable generation sites to load centers^1^. Specifically, a microgrid energy systems connects renewable energy resources, such as solar, wind, hydro, geothermal, and biomass, and the potential for renewable integration is immense when considering both grids-connected and islanded applications communities. Further, energy systems processes capture customer and grid power usage data utilizing digital technologies, measures energy transaction process data, communicates the data via fast and efficient communication devices, stores energy transaction process data while communicating between energy metering and distribution, and actively process grid management via decentralized control^2,3^. However, utility-based renewable energy microgrid energy systems experience significant technical and economic vulnerability due to the intermittent and environmental supply nature of the technology intruding on parts of the overall power converter stage, a lack of defined regulatory rules, elements of network operation, management strategy, and complex responses to address issues of local and wide-scaled conversions along a centralized or decentralized overall microgrid systems^2,3^.

In summary, the main contributions of this research work are:

- Development and implementation of the Bald Eagle Search (BES) algorithm for the optimal allocation of photovoltaic (PV) and battery energy storage systems (BESS) in the distribution network to minimize operating costs, power losses, and enhance voltage profiles.
- Comparison of the proposed algorithm with other existing optimization techniques, such as Genetic Algorithm (GA) and Whale Optimization Algorithm (WOA), for various operating conditions to establish its superiority.
- Investigation of the PV-only and hybrid PV-BESS systems to identify the technical and economic advantages of integrating batteries.
- Validation of the proposed algorithm on standard distribution networks with statistical analysis to ensure the feasibility of the optimal results.

The remainder of this paper is organized as follows. Section  2 provides a review of the relevant literature, highlighting previous studies and identifying existing research gaps. Section  3 presents the problem formulation, including the system model, problem definition, and underlying assumptions. In Sect.  4, the Bald Eagle Search (BES) algorithm is introduced, along with a detailed description of its mechanism and its adaptation to the proposed problem. Section  5 reports the simulation results that demonstrate the performance of the algorithm. Section  6 validates the proposed approach through comparisons with benchmark methods. Finally, Sect.  7 concludes the paper and outlines directions for future research.

## Literature review

### Overview of hybrid microgrid optimization techniques

The optimization of hybrid systems within microgrids has been widely researched from different perspectives and to meet various objectives. Examples of this can be seen in studies which aimed to minimize power losses by developing an optimal location and size for of the renewable distributed generation and battery storage; the authors employed a variety of intelligent optimization techniques. More recent studies are investigating the use of optimization methods - for example Coyote algorithm - to improve the power quality indices of the microgrid and the voltages for different aspects of grid problems; other studies investigated the development of combined photovoltaic and battery systems - using gyri-focusing approach - to minimize losses and add cost savings to the distribution feeder-specific studies look to add a geographic context to analysis (i.e. Switzerland); there are also studies addressing operational challenges of their hybrid system, outlining that it may not be a perfect solution for minimizing losses but may nonetheless be useful for enhancing voltage profile aspects; however overlooks the key aspect of optimal placement^4^.

Moreover, researchers have investigated sophisticated optimization strategies for the selection of battery capacities by including variations in load and distributed generation output. For example, genetic algorithms have been used to minimize power losses and voltage deviations as a bore. Particle swarm optimization has been utilized to increase the capability of power transfer through the optimal size of storage units in microgrids, which helps improve loading limits of lines. Full techno-economic analyses have been conducted to study the impact of different battery storage technologies and control strategies on the capacities of photovoltaic (PV) systems in distribution networks. Optimal capacity storage systems have been characterized using local marginal pricing and genetic algorithms, both of which reduce outages and determine PV panel sizes. Additionally, Equilibrium Optimizers and such models inform the design of hybrid systems, with the intention of achieving reduced losses and greater voltage stability. All approaches showed substantial improvements where hybrid systems were optimally placed and sized^4,5^.

### Characteristics and topologies of distribution networks

Recently, the power system has evolved into microgrids, oases of self-governing entities forming an interconnections of distributed generation units, loads, and energy storage units with an ever-increasing energy efficiency. Meanwhile, the inconsistency of renewable generation introduces power imbalances that must be effectively managed through microgrid energy management, offering possibilities for many new approaches presented in the literature, which include an effective energy management and control mechanism of an autonomous microgrid consisting of a photovoltaic (PV) system, wind turbine (WT) and battery energy storage system (BESS), in which a controller designed to a high standard maintains the hybrid power system stability, which lends itself to the coverword of benefits all, implying an optimal location, an optimal sizing, or an optimal use of distributed generation units^6^. The process of electrical power delivery from generation to utilization is complicated by a system composed of generation equipment such as power plants and substations, transmission lines and substations, and distribution systems that transport electricity to end-users, which in many cases have radial structures and are susceptible to voltage drops, instability, and losses in power due to the high R/X ratio of the lines, especially in critical loading situations; this requires attention to voltage regulation, stability, and loss minimization in their design and operation.

The design and operation of distribution networks are influenced by various but interrelated factors. Societal reliance on an electricity supply with reliability and financial consequences of power outages are some of these interrelated factors. However, the high-quality power—i.e. stable voltage and low harmonic distortion—are also needed along with safety for end-users and utility workers. All of these issues with technology and safety will be traded with regard to economic factors including reducing losses in the network as well as economic efficiency with power quality or best investment in capital infrastructure. Additionally, there are practical issues such as availability of equipment, placement of transformers and substations as well as existing regional policies that affect the electric power industry. In some cases, these policies may favour rapid expansion of the distribution network significantly above strictly applied technical standards due, in many cases, to financial issues^7^.

The design and operation of distribution networks is a function of multiple factors that are related but distinct from each other. For example, there is an expectation by society of a certain reliability from an electricity supply that includes a consideration of the financial consequences of a power interruption and/or defects in supply. Then there is also a requirement of power quality, meaning voltage stability and little harmonic distortion to name just two; and this is employed with safety considerations for the customer using the electricity and for the utility staff. All of these technological issues and safety considerations will be traded around an economic view, that includes attempts to reduce losses in the network as well as a view of economic efficiency of power quality or best investment of capital into fixed assets. Also of consideration is equipment availability and the location of transformers and substations; as well as the policies of geographical regions that govern the electric utility industry. In some scenarios, that policy may support the rapid extension of the distribution network well beyond strict technical standards, usually including a financial component in the justification for this strategy^7^.

Considering the voltage levels of the power system, the geographical conditions of the region, the weather conditions, and the load concentration in the area, different topologies of the distribution system can be used to supply electrical energy to the subscribers. Electrical energy distribution networks can have any type of topology, but in standard form, three general types of topology, radial feeders, loop feeders, and network feeders can be introduced for distribution networks. In radial feeders, the circuit is drawn from the substation to the distribution transformers and continues to the end of the network, and all loads are only fed from one side. Among the advantages of this system, the simple design and low construction cost of this type of feeder can be mentioned, but the main disadvantage of radial feeders is the outage of the defective part to the end of the network in this type of topology, which increases the costs of unsold energy to the subscribers and as a result, reduces the reliability indicators in the distribution system and dissatisfaction of the subscribers^7,8^. To solve this problem in recent years, alternative maneuver lines have been used to supply power to the unpowered parts by nearby networks. To improve the reliability indicators of distribution feeders, they can be designed in a loop form. In this topology, the medium voltage network is fed after the sub-transmission substation and after passing through the distribution substations, it returns to the same substation. In this topology, if an error occurs on the feeder, the circuit breakers immediately operate and separate the damaged part from the network. The rest of the network sections that are not affected by the error are fed from another path of the network. This process in distribution feeders is called network reconfiguration. The advantages of this topology compared to the radial topology can be mentioned as follows: the loop feeder has fewer outages compared to the radial feeder, the loop feeder does not need to use maneuver lines, and in the loop feeder, there is no concern about insulation failure in the lines. The most complete and the most complex topology of distribution networks is the network topology. In this structure, each of the distribution substations is connected to several other distribution substations at different distances. In the network topology, one or more sub-transmission busbars can be used to feed the network. This type of feeder creates the highest level of service quality for the subscribers. in this topology, in case of an outage of the sub-transmission busbar, the subscribers of that feeder will not be without power and will be fed from the adjacent busbars. due to the high economic costs, the complexity of the design and coordination, as well as the problems of operation and, on the other hand, the difficult control of load distribution and the performance of protection equipment, this topology is less used in the distribution network^9,10^.

### Power losses in electrical distribution networks

After being produced in power plant units and passing through transmission, sub-transmission and distribution lines, electrical energy reaches consumers. A large amount of energy is lost and converted into losses due to various reasons during the transmission of electrical energy from production to consumption. The history of the topic of energy loss dates back to the age of the electricity industry in the world, and of course the loss of electrical energy and its problems are extensive, but what has caused this problem to continue to be a current topic in electrical engineering communities is the negative economic consequences and high costs that must be paid due to losses in distribution feeders. therefore, it is necessary to pay more attention to the issue of energy loss in the power system, which is an undeniable and obvious necessity. According to the statistics provided by Tavanir, the amount of energy loss is 23.3% of the total electricity levels in the country, including production, transmission and distribution, and the distribution system accounts for approximately 55% of the energy loss. The amount of electrical energy loss in transmission and production levels is small. Electrical energy loss, which is proportional to the square of the current passing through the system components, is much higher in peak consumption hours than the average electrical energy loss. This means that in the worst case, that is, during peak hours, we will also have the highest amount of electrical energy loss. By examining the distribution feeders, it is determined that by considering measures, the amount of electrical energy loss can be greatly reduced. In general, the factors affecting the loss of electrical energy in distribution networks are very numerous, and they can be divided into two categories: technical network loss and non-technical loss in the power system. In non-technical loss, issues such as unauthorized use of electricity and energy theft in an obvious way, unauthorized use of electricity in a hidden and covert way and tampering with meters, removing them from the circuit, not accurate reading of reading officers and improper functioning of meters and not calibrating them), and errors in calculations can be mentioned^11,12^.

In the first place, a number of technical factors may also lead to losses of energy within the electrical power distribution networks. They include the losses associated with inherent and internal losses of electrical equipment, losses resulting from weather, energy losses caused by inaccuracies in measurements in meters and instruments, and many others. Moreover, energy losses are contributed by factors such as low power factor, unbalanced loading of the network, and poor quality of appliances and electrical components. Furthermore, factors such as the proper design of a system, loose connections in distribution feeders, and poor practicing of grounds contribute to overall losses. The use of long distribution lines to deliver power to remote villages, frequently due to social needs, can also be another source of a reserve of inefficiency. In addition, when there is insufficient planning to start and shut down power plants adequately, it results to an overflow of associated energy losses of power. Other losses also occurs when there is poor consideration in the rehabilitation of outdated distribution infrastructure, poor identification of location and capacity for 20 kV substations, and the lack of systematic or holistic approach to the network planning. Ultimately, a low consumer power factor, insulator and bushing contamination, high harmonic distortion, and high reactive power is some of the other major causes of technical losses. Other contributing factors to the wasted energy in distribution networks, in addition to what has been mentioned, are management incompetence, underdevelopment of staff through limited training and professional development, the dominance of master craftsmen in the formerly public utility, etc.

The important factors in intensifying the loss of electrical energy are as follows: lack of attention to the necessity of optimizing and reconstructing worn-out distribution networks, non-optimal and unprincipled selection of the installation location and capacity of 20 kV substations, the prevalence of the master craftsman culture, the low power factor of subscribers, the widespread use of low-section wires, the lack of proper load management, the lack of voltage range stabilization in the distribution feeder, the lack of a comprehensive network reform plan, the lack of coordination and balance between production and consumption, the use of iron alloy connections instead of aluminum connections. To reduce the loss of electrical energy in the distribution network, in addition to paying attention to the parameters that intensify the wasted electricity and trying to eliminate or reduce them, different methods in the field of loss reduction in distribution systems are used, the most important of which can be mentioned as follows: using capacitors in the network, using harmonic filters, using conductors with higher cross-sectional area, using distributed generation sources, network reconfiguration, load balancing, improving the power factor of household and commercial subscribers, voltage regulation by means of tap changers. among the mentioned procedures, installing distributed generation sources along the distribution feeder is one of the most widely used actions in distribution companies. If distributed generation sources are installed in a favorable location and with an optimal amount in the system, they can improve the voltage range and reduce the loss of electrical energy. The traditional size of electricity networks has always been based on the absence of active sources of electrical energy production in distribution feeders. With the increase in the number of installed size of these sources in the distribution system and due to the existence of different technologies in the construction and use of these sources, the study of the properties of these types of sources at the time of connection and their parallel operation with electrical networks is very important. There are two theories about the description of the existence of distributed generation in the distribution network. Some believe that distributed generation should supply all the electrical power needed by the distribution network, and in the operation of these networks, there is no need for transmission lines and large power plants. However, others believe that distributed generation should meet a certain part of the locally needed power. depending on the characteristics of the network and the type of distributed generation sources, it can have positive or negative results^13,14^. Distributed generation sources in distribution feeders, in addition to generating electrical energy, have different properties that are very useful for the distribution feeder. Considering the low voltage level of the end of the system, the installation conditions of distributed generation units in the distribution network are possible. to evaluate distributed generation, we need to investigate the benefit of distributed generation^15^.

### Analytical modeling and performance indices of DG systems

Distributed generation (DG), in^16^ addition to producing electrical energy, has various properties that are very useful for distribution feeders. Given the low voltage level of the system terminals, the installation conditions of DG units in the distribution network are possible, and to evaluate these productions, we need to investigate their benefit, which includes reducing the ohmic losses of the lines.

Incorporating distributed generation (DG) into distribution systems can greatly reduce energy flowing through the distribution lines, and, therefore, energy losses. This is due to network losses being directly proportional to the square of current flowing through the lines; thus, the lower the current, the lower the losses. Moreover, DG devices can provide load compensation, thus, reducing voltage drops across the network. During peak load conditions, these issues become apparent. Reduced voltage drop implies lower energy losses and improved performance and efficiency during normal operations and emergencies (e.g., faults and failures). Without DG, energy loss increases with distance of purchase of power from distant resources creating larger voltage drops and losses. Studies have shown that energy losses in a distribution network can be optimized as much as 51% including consideration of network configuration and optimal placement of DG units. Therefore, DG location requires careful consideration to optimize utilization of DG sources.

On the other hand, distributed generation leads to the release of line capacity, which is usually done in two ways: distributed generation reduces the line current by improving the voltage profile, and also distributed generation reduces the current passing through the line by supplying part of the load current. Finally, the line drop index (LLRI) is also defined^17–19^ as:

1 $$LLRI = \frac{{LL_{{W/DG}} }}{{LL_{{WO/DG}} }}$$

where LL_w/DG_ and LL_wO/DG_, respectively, represent the line losses in the cases of utilizing DG and not utilizing it in the network. As can be seen from Eq. (1), a lower LLRI indicates a better voltage profile and reduced line current, which in turn reduces line losses and improves overall network efficiency.

In addition to their role in generating electrical energy, distributed generation (DG) has numerous properties that are beneficial to distribution feeders. Since the system terminals are at a distribution voltage level, it is practical to install DG units in the distribution network. In evaluating these DG systems, their benefits must be investigated, with one of these being the reduction of ohmic line loss. The introduction of DG into a distribution network will, in a sense, reduce the current in the lines, and hence the power loss in the networks. Since network loss is a function of the square of current flowing in the wires, load compensation through DG sources can reduce line ohmic loss, on a volumetric basis.

This affect is amplified during peak load periods. The reduction of these losses also improves efficiency^18,19^. The advantages of distributed generation (DG) in loss reduction are even more valuable in events of emergencies, such as a system fault or outage. In those scenarios, the percentage of electric power provided by those farther generators increase leading to the result, once to increase the voltage drop and losses. It has been reported that losses can be lowered as much as 51% depending on the arrangement of the DG and DG types. The information highlights the need for a systematic process for evaluating the location of DG in the hopes of increasing the benefits of DGs. Additionally, the DG can free the line capacity, which can be done in two ways, 1- By enhancing the voltage profile of the line current, which reduces load current by DG, or 2- The DG also supplies a portion of a load current which also results in an effective current decrease in the line. Meta-heuristic optimization approaches have also demonstrated effectiveness in hybrid microgrid capacity allocation and operational optimization, leading to improved economic performance and system efficiency^20^. To position this scenario mathematically, let LLW/DG represent line losses with DG facility operation, and let LLWO/DG represent line losses without DG facility operation^13,15^.

2 $$\:L{L}_{W/DG}=3\sum\limits_{I=1}^{M}{I}_{A,i}^{2}R{D}_{i}$$

  

3 $$\:L{L}_{WO/DG}=3\sum\limits_{I=1}^{M}{I}_{L,i}^{2}R{D}_{i}$$

  

In Eqs. (2) and (3), *R* is the line resistance in terms of *Di*, pu/km, the length of the energy distribution line, and *M* is the number of lines in the system, and *I*_*A, i*_ is the per-unit current of the lines in the system, part of the power of which is supplied by DG, and *I*_*L, i*_ is the per-unit current of the lines in the system in which there is no DG. Now we can have the following interpretations:

1. *LLRI* < 1: The presence of DG has increased the loss of lines.
2. *LLRI* = 1: The presence of DG has no effect on the loss of lines.
3. *LLRI* > 1: The presence of DG has led to a reduction in the loss of lines.

It is clear that the best place to install DG from the point of view of this index is the place, where the *LLRI* has the lowest value. Using these definitions, the percentage reduction in line losses due to DG integration can be expressed as^19^:

4 $$\:Loss\:Reduction\left(\%\right)=\:\frac{L{L}_{WO/DG}-L{L}_{W/DG}}{L{L}_{WO/DG}}\times\:100$$

This formulation allows the quantification of DG impact on network efficiency, where higher values indicate more effective loss reduction.

Distributed generation resources can increase the voltage level of the distribution network by reducing the flow of active and reactive current in the distribution lines, which improves the voltage level of the network, and these systems can be used as a reactive power compensator in addition to supplying the active power of the network. In general, the problem of voltage drop in the distribution network occurs when the network is under peak load or in an overload state, in which cases the voltage drop can be improved by reducing the current flow in the lines. One of the many elements used in this section is the capacitor. By injecting reactive power into the network, the capacitor reduces the reactive current of the lines and, in the end, the voltage drop is reduced. However, if we replace the capacitor with a distributed generation system, in addition to reducing the reactive current, we will also reduce the active current of the line. In other words, a distributed generation unit can, in addition to absorbing and producing reactive power, also produce active power. In practice, active power has a large direct effect in regulating the voltage drop in the network, because most of the network loads are made up of inductive impedances of lines, transformers, and inductive loads, which create the main part of the voltage dro. The voltage profile improvement index (*VPII*) is defined as follows^21^:

5 $$VPII = \frac{{VP_{{W/DG}} }}{{VP_{{WO/DG}} }}$$

where, *VP*_*W/DG*_ and *VP*_*WO/DG*_ represent the voltage profile indices with and without DG, respectively. The voltage profile index is defined as follows:

6 $$VP = \sum\limits_{{i = 1}}^{N} {V_{i} L_{i} K_{i} }$$

In the above relations, *V*_*i*_ is the voltage magnitude in per unit, *L*_*i*_ is the load in per unit, and *K*_*i*_ is the weighting factor in the *i*-th bus, and *N* is the total number of buses in the distribution system. According to the above relations, the following three cases each have a specific meaning:

1. *VPII* < 1: The effect of DG on the network voltage profile was negative.
2. *VPII* = 1: DG had no effect on the voltage profile.
3. *VPII* > 1: The effect of DG on the network voltage profile was positive.

The index can be used to determine the best location of distributed generation (DG) units to manage the voltage levels in the network. The best application of distributed generation is when the index has the highest value. Distributed generation improves the network reliability in two ways: While at the same time releasing line capacity; and while working as a reserve or backup power. The line has greater capacity once the line capacity is released with distributed generation, and the line is capable of moving a part of a full load of the faulted line; in other words, a distributed generation source allows load transfer away from the faulted line onto available line capacity.

The second aspect of improved reliability occurs when the main grid is not available and the DG unit is supplying power directly to the local load. This allows the outage time for the customer to be decreased and increases electric reliability. In addition, when power is produced under both base load and peak load situations, DG adds to the general availability of the power system. Therefore, DG increases reliability along with several other reliability indices. Additionally, because DG reduces fault recovery time in the network, it also improves safety indices. Even though the impact of voltage drop isn’t directly reflected in reliability indices, it can increase service outage time, since it causes equipment to age faster and increases temperatures. As a result, DG improves reliability and operational safety of the distribution network by reducing voltage drop^21^.

Reducing the energy transmission cost: availability to the place of consumption is one of the very important values of distributed generation, this method leads to a significant reduction in the transmission cost, reducing the installation and operation time of the unit: the installation location of distributed generation units is much less compared to large and centralized power plant units, in this way, these units can be put into operation in a short time, reducing investment at peak load: by constructing and using distributed generation sources at the peak of the network, the required periodic needs can be met, on the one hand, the investment of distributed generation is much less compared to traditional centralized power plants, realizing the idea of privatization in the electricity industry: in traditional electricity systems, only large investors in the country can participate, but with these small-scale power plants, small investors can also enter this field, increasing the life of the power system: reducing the loss of electrical energy and reducing the peak load values of the system can lead to an increase in the life of the devices, these cases lead the equipment to operate in a more suitable temperature range, as a result, their lifespan will increase, achieving all the mentioned goals is very difficult and challenging from a technical point of view, because this method requires having distributed generations with high reliability, proper controllability and having the appropriate size and installation location in the distribution feeder^22^.

### Recent advances in battery energy storage system optimization

In recent years, battery energy storage systems (BESS) have emerged as an essential element of renewable-rich power systems because of their capability to improve the flexibility, reliability, and economic viability of distribution networks. Various efficient optimization techniques have been proposed that target the sizing, placement, and control of BESS in combination with photovoltaic (PV) integration.

For instance, ref^23^. proposed a holistic BESS sizing and placement algorithm that takes into account dynamic pricing and load changes to optimize costs in the distribution network. This reference emphasizes the need to align storage operation schedules with pricing tariffs for optimal costs. Furthermore, a comprehensive review in Batteries assessed cutting-edge battery management and optimization approaches, focusing on lifecycle aspects and control methods for enhanced storage performance and life with high renewables integration^24^. A hybrid metaheuristic technique for the joint optimization of PV and BESS is proposed in Ref^25^., which has been shown to have better convergence and solution quality than the conventional GA and PSO algorithms. Optimal allocation of BESS with demand response, as shown in^26^, has been found to reduce network losses and voltage deviations.

A hybrid optimization method for BESS allocation and sizing was proposed in^27^. A collaborative scheduling approach for integrated PV-EV-BESS systems was proposed in^28^, which demonstrated the efficacy of peak shaving and valley filling. The economic operation of BESS in microgrid-connected systems was discussed in^29^, which emphasized the economic benefits of BESS in terms of cost saving and improvement of grid reliability. Furthermore, Ref^30^. proposed a multi-objective EMS for hybrid PV-wind-BESS systems, which reiterated the importance of BESS in improving the performance of renewable energy systems.

These studies collectively confirm that BESS is an essential component in loss reduction, voltage regulation, and cost minimization; nevertheless, the optimal sizing and placement of PV-BESS in radial distribution networks with the simultaneous objective of loss minimization while improving voltage profiles has not been adequately investigated. The present study addresses the gaps by using the Bald Eagle Search (BES) algorithm for optimal PV-BESS sizing and placement in a radial distribution network, which shows better convergence, lower power losses, and better voltage profiles than GA and WOA algorithms, offering a reliable and effective solution for the optimization of hybrid PV-BESS systems in distribution networks.

**Table 1:** Comparative review of PV and BESS integration studies in distribution networks.

Study	PV	BESS	Method	Objective Type	Voltage Improvement	Loss Reduction	Limitation
Baran & Wu (1989)	✖	✖	Analytical	Single	Low	Moderate	No DG integration
Saboori et al. (2015)	✖	✔	GA	Single	Good	Good	No PV integration
Verma & Padhy (2021)	✔	✖	OPF	Multi	Moderate	Moderate	No storage modeling
Pompern et al. (2023)	✔	✔	Metaheuristic	Multi	Good	Good	Single-algorithm study
Wichitkrailat et al. (2024)	✔	✔	COA	Multi	Good	Good	Limited benchmarking
Shaier et al. (2025)	✔	✔	EA	Multi	Good	Good	High complexity
Proposed Work	✔	✔	BES, GA, WOA	Multi	High	High	Comprehensive comparative framework

The comparative analysis presented in Table 1 shows that although significant progress has been made in the optimal allocation of PV and BESS in distribution systems, most existing studies either focus on single-method optimization or lack a comprehensive comparative evaluation of multiple metaheuristic algorithms under identical system conditions. In addition, several works consider PV-BESS integration; however, they do not simultaneously evaluate key performance indicators such as operating cost, power loss reduction, and voltage profile improvement across multiple scenarios. Therefore, a clear research gap exists in developing a unified and systematic comparative optimization framework for coordinated PV and BESS planning in distribution networks. To address this gap, this study proposes a BES-based framework compared with GA and WOA for optimal allocation and sizing of PV and BESS in a 69-bus distribution system.

## System modeling, methodology, and problem formulation

In the last two decades, one of the most important issues for engineers in the field of distribution networks has been the reduction of electrical energy losses in the form of losses and the improvement of power quality indicators, reliability and supply of electricity needed by subscribers at a lower cost for consumers. The use of distributed generation sources in power distribution feeders and their optimal location can reduce electrical energy losses in the network, improve power quality and voltage stability, reduce outages and improve reliability and peak shaving in the network^31^. The research work is relevant to the optimal sizing and location of photovoltaic (PV) power and battery energy storage systems (BESS) in a 69-bus radial distribution network. The primary aim of the research work is to ensure that the total cost of operation of the system is minimized. In this paper, the reduction of operating costs and improvement of the voltage profile in a standard radial distribution network using photovoltaic distributed generation has been investigated. In this section of the paper, the distribution feeder under study is first discussed and then the objective function and constraints of the problem are stated.

### Test system description and data specification

**Fig. 1:**
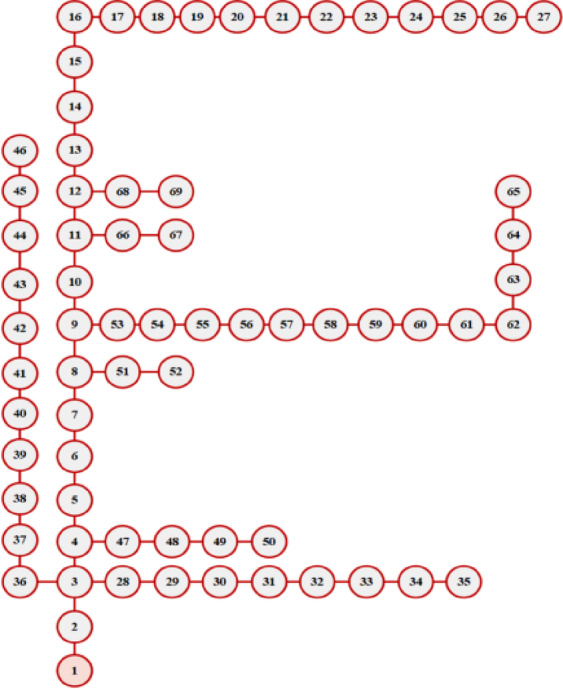
Structure of the 69-bus standard distribution network studied.

The studied distribution network is a radial feeder with 69 buses, incorporating distributed photovoltaic (PV) generation and optional battery energy storage systems (BESS). The system configuration, line impedances, and bus connectivity are based on the standard IEEE 69-bus test feeder^32^. The structure of this network is shown in Fig. 1. The load at each bus is represented using a ZIP model (Z: 30%, I: 20%, P: 50%), which reflects realistic voltage-dependent load behavior. The network is assumed to operate under balanced conditions.

The solar irradiance values are extracted from the Typical Meteorological Year (TMY3) dataset provided by NREL^33^, which gives hourly solar irradiance and temperature data typical for a year at the chosen location. The PV systems are connected to buses that are determined through optimization. The BESS systems are represented as controllable storage resources that can charge and discharge depending on the network conditions for peak shaving, voltage support, and loss minimization. The results are obtained for a typical day of 24 h. Table 2 shows the summary of distribution feeder and load data.

**Table 2:** Summary of distribution feeder and load data.

Parameter	Value/Description
Number of buses	69
Base voltage	12.66 Kv
Peak total load	3.7 MW, 2.5 MVar
Load type	ZIP (Z: 30%, I: 20%, P: 50%)
Line type	Overhead lines and underground cables
Line resistance (R)	0.092–0.5 Ω/km
Line reactance (X)	0.047–0.25 Ω/km
Maximum bus voltage	1.05 p.u.
Minimum bus voltage	0.95 p.u.
Solar irradiance dataset	NREL TMY3, Typical Meteorological Year^33^
PV array locations	Selected via optimization (PV1–PV8)
BESS locations	Selected via optimization (BESS1–BESS8)

**Fig. 2:**
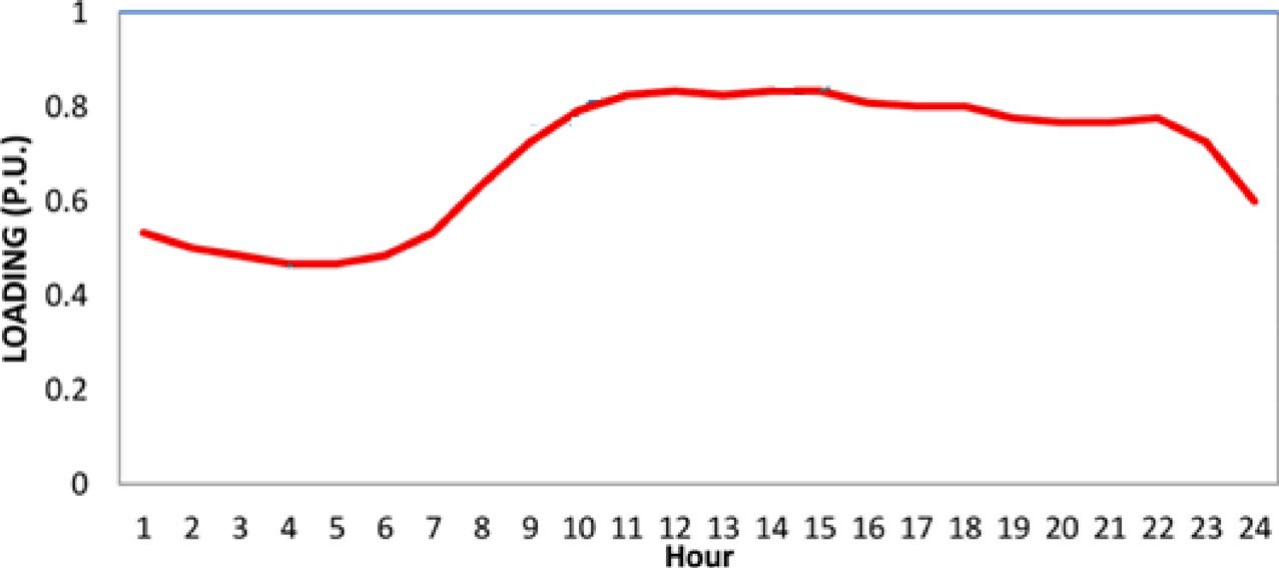
Load curve of the distribution system under study.

The bar graphs in Fig. 2 show the amount of changes in electrical energy consumption and the network load profile in 24 h. The minimum electrical energy required in the feeder is about 0.42 per unit at 4 am, and the maximum amount of network load is also determined at 2:00 PM and is equal to 0.82 per unit.

**Fig. 3:**
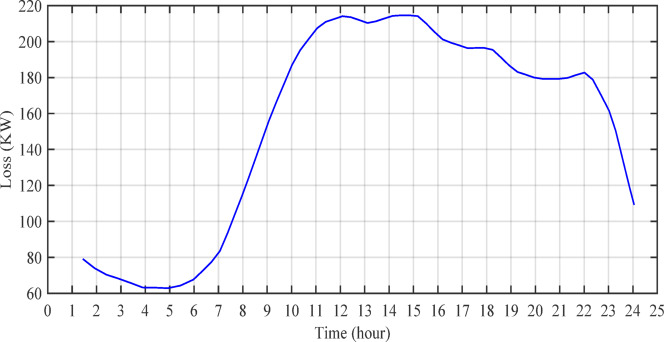
Electrical energy losses in the system under study.

The nominal load of this network is about 4.4 MVA, and the total reactive power of the loads in the feeder is about 2.3 MVAR. The power factor of this feeder is also considered to be 0.85 lagging. Figure 3 shows the amount of electrical energy loss in the distribution lines in 24 h in the absence of photovoltaic distributed generation sources.

**Fig. 4:**
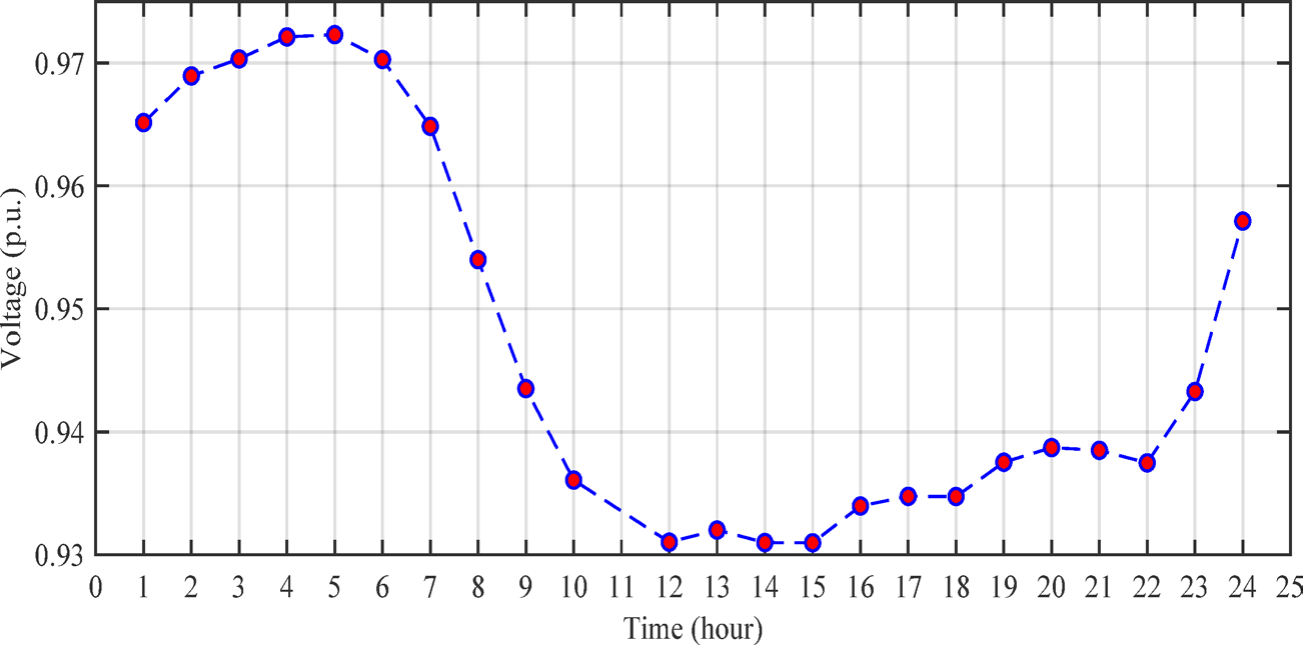
Grid voltage curve in 24 h.

In this paper, a radial feeder with a specific structure is investigated. Based on the bar graphs, the amount of electrical energy consumption and the network load profile in 24 h are determined. In addition, the highest and lowest amount of electrical energy required in the feeder is specified, and according to the nominal load, the total reactive power of the loads and the power factor are also calculated. In this study, the amount of ohmic losses in the network is also calculated, and the maximum amount of electrical energy loss is determined. The voltage deviation index is also calculated for this feeder, and its value before the installation of photovoltaic sources is shown in Fig. 4.

**Fig. 5:**
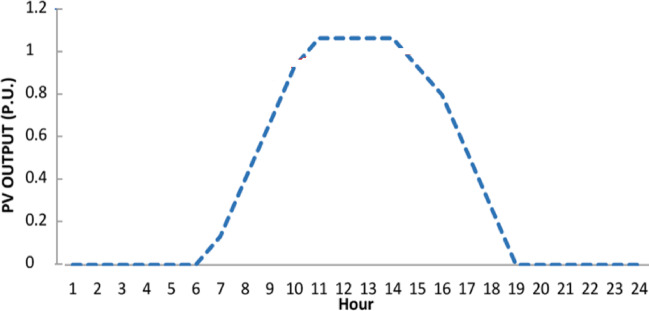
Solar radiation intensity in 24 h of study.

In addition, in this study, it has been determined that the amount of voltage deviation in peak load hours is more than other hours. Finally, to improve the voltage deviation index and reduce ohmic losses, the installation of photovoltaic distributed generation sources and the use of intelligent systems to control the load and optimize network performance have been proposed. The amount of energy production from solar sources strongly depends on the solar and wind radiation in the region. For this reason, in the simulation period, there is a need to have the minutes of solar and wind radiation accurately. Figure 5 also shows the daily solar radiation intensity. According to the solar radiation intensity in the region, from 7:00 AM to 6:00 PM, there are conditions for receiving solar power in the network.

### Objective function and constraints

The paper is to reduce the cost of a distribution feeder by re-arranging the switches in the distribution feeder and determining the optimal capacity of distributed generation sources using optimization algorithms. For this purpose, it is necessary to determine a suitable objective function to consider all the operating costs of a distribution feeder. Accordingly, the overall optimization problem is formulated as a multi-objective cost minimization problem^23,25^:

7 $$Minimize~F={C_{BES}}+{C_{PV}}+{C_{Loss}}$$

where C_PV_ represents the total cost of photovoltaic systems, C_BES_ denotes the total cost of battery energy storage systems, and C_Loss_ is the cost associated with energy losses in the distribution network. In the objective function represented by Eq. (7), the total cost of operation takes into consideration both BESS and PV systems. In the case of BESS, the cost of operation considers cycling costs, efficiency losses, and any maintenance costs that may be incurred during operation. In the case of PV systems, the cost of operation considers maintenance costs as well as degradation costs over the life of the system^23,24^.

8 $${C_{PV}}=Cos{t_{PV}}CRF{}_{{PV}}$$

9 $${C_{BES}}=Cos{t_{Bat}}.CRF{}_{{Bat}}$$

The capital recovery factor (CRF) is calculated as^23,24^:

10 $$CRF{}_{{PV}}=\frac{{r{{\left( {1+r} \right)}^{L{T_{PV}}}}}}{{r{{\left( {1+r} \right)}^{L{T_{PV}}}} - 1}}$$

11 $$CRF{}_{{Bat}}=\frac{{r{{\left( {1+r} \right)}^{L{T_{Bat}}}}}}{{r{{\left( {1+r} \right)}^{L{T_{Bat}}}} - 1}}$$

where, *CRF* is the capital recovery factor, where *r* is the discount rate and LT is the lifetime of that equipment. In addition, *Cost*_*PV*_ is the cost of each installed kilowatt of photovoltaic sources. This cost includes initial investment, maintenance, and cleaning costs of the photovoltaic array surface. *Cost*_*Bat*_ is the cost of each installed kilowatt of battery storage. In addition, the cost of losses calculated based on the total energy lost over a period multiplied by the cost of energy per kWh^13,25^:

12 $${C_{Loss}}=\sum\limits_{t} {\sum\limits_{i} {R(i)} } \times I(i,t) \times CE(t)$$

In relation, *R*(*i*) and *I*(*i*) are the resistance and current passing through the *i*-th branch, respectively. In addition, *CE*(*t*) is the price of electricity at time *t*. The price of electricity is different at different hours of the day, and in this paper, the simulations are performed based on time-of-use (TOU) prices. This method is the most common type of pricing that most countries use. In this method, the hours of the day are divided into several time periods, and a different price is considered for energy in each period. The simplest type of TOU has two time periods, peak and off-peak, and sometimes mid-peak periods are also included in this classification. The prices of the mid-peak period are higher than the off-peak periods and lower than the peak periods. In this study, the investment cost of PV systems is set to CostPV = 900 USD/kW, the investment cost of BESS is CostBES = 400 USD/kWh, the project lifetime is assumed to be 20 years, and the annual discount rate is 8%. The electricity price is considered as 0.12 USD/kWh, and the capital recovery factor (CRF) is calculated accordingly based on the project lifetime and discount rate.

When installing photovoltaic sources, some limitations must be considered. All buses must be in the allowable range of 0.9 per unit to 1.1 per unit, and the balance between electricity generation and consumption must always be maintained^21,22^.

13 $${{\rm P}_{grid}}+{P_{DG}}={{\rm P}_{demand}}+{P_{Loss}}$$

14 $${Q_{grid}}+{Q_{DG}}={Q_{demand}}+{Q_{Loss}}$$

In the above relations, *P*_*grid*_, and *P*_*DG*_ are the active power received from the national electricity grid, the active power generated by photovoltaic distributed generation sources, and the active power consumed by the load, respectively, and *Q*_*grid*_, and *Q*_*DG*_ are the reactive power provided by the national grid, the reactive power generated by photovoltaic sources, and the reactive power required by the load, respectively, and *P*_*Loss*_ and *Q*_*Loss*_ are the active and reactive power losses in the system, respectively. It is obvious that by using an inverter in the photovoltaic system, it is possible to generate reactive power for it. In addition, one of the limitations considered for the distribution system with photovoltaic sources is the limited capacity of power transfer from the lines. Therefore, the power passing through the lines after the rearrangement should not exceed the maximum allowable thermal capacity^21,22^.

15 $${P_{Line}}(ij) \leqslant P_{{Line}}^{{Max}}(ij)$$

In the above relation, is the maximum allowable power passing through the distribution lines. One of the limitations considered for distributed generation is that at any hour, the active and reactive power generated by distributed generation sources must be within its capacity range^21,22^.

16 $$\begin{gathered} {\rm P}_{{DGi}}^{{\hbox{min} }} \leqslant {{\rm P}_{DGi}}(t) \leqslant {\rm P}_{{DGi}}^{{\hbox{max} }} \hfill \\ \mathop Q\nolimits_{{DGi}}^{{\hbox{min} }} \leqslant \mathop Q\nolimits_{{DGi}} (t) \leqslant \mathop Q\nolimits_{{DGi}}^{{\hbox{max} }} \hfill \\ \end{gathered}$$

Where P_DG_, Q_DG_ are the active and reactive powers of distributed generation. In this case, the minimum amount of distributed generation is considered to be zero and the maximum is two megawatts, and the power factor of distributed generation sources is less than the defined allowable power factor. In total, the minimum power factor for distributed generation operation is considered to be 0.8^21,22^.

17 $$pf_{{DGi}}^{{\hbox{min} }} \leqslant p{f_{DGi}}(t)$$

### Modeling assumptions for PV and battery energy storage systems

To ensure transparency and reproducibility of the proposed framework, the main modeling assumptions adopted for PV and BESS systems are summarized as follows. The output power of each PV unit is modeled as a function of the solar irradiance and the rated capacity of the PV array, expressed as^4,34^:

18 $$\:{P}_{PV}\left(t\right)={P}_{PV,i}^{rated}\times\:\frac{G\left(t\right)}{{G}_{STC}}$$

where, $\:{P}_{PV}\left(t\right)$ is the output power of the PV unit at bus *i* and time *t* (kW), $\:{P}_{PV,i}^{rated}$ is the rated capacity of the PV unit at bus *i* (kW), G(t) is the solar irradiance at time *t* (W/m²), G_STC_ is the standard test condition irradiance (1000 W/m²). It is assumed that PV systems are equipped with grid-connected inverters capable of operating at a controllable power factor within the range of 0.8 to 1.0. Reactive power support from PV inverters is allowed within their apparent power limits.

The battery system is modeled using a state-of-charge (SOC) based formulation. The SOC of each battery at time *t* is updated as^23,24^:

19 $$\:{SOC}_{i}\left(t+1\right)={SOC}_{i}\left(t\right)+\frac{{\eta\:}_{c}{P}_{ch,i}\left(t\right)-\frac{1}{{\eta\:}_{d}}{P}_{dis,i}\left(t\right)}{{E}_{BES,i}^{rated}}$$

where,$\:\:{SOC}_{i}\left(t\right)$ is the state of charge of the battery at bus *i* at time *t*, $\:{P}_{ch,i}\left(t\right)$ and $\:{P}_{dis,i}\left(t\right)$ are the charging and discharging powers (kW), $\:{\eta\:}_{c}$ and $\:{\eta\:}_{d}$ are the charging and discharging efficiencies, $\:{E}_{BES,i}^{rated}$ is the rated energy capacity of the battery (kWh). The SOC is constrained as^23,24^:

20 $$\:{SOC}_{min}\ll\:{SOC}_{i}\left(t\right)\ll\:{SOC}_{max}$$

where $\:{SOC}_{min}$and $\:{SOC}_{max}$ are set to 20% and 90%, respectively, to prevent deep discharge and overcharging. It is assumed that:

- Batteries cannot charge and discharge simultaneously.
- Battery degradation effects are neglected.
- Battery efficiency is constant.
- Self-discharge is ignored.
- BESS is used only for energy arbitrage and loss minimization.

In this study, a Lithium-ion BESS is considered, with the following assumptions made in the modeling: a round-trip efficiency of 90%, an SOC range of 20% to 100%, and optimized capacities. These assumptions are made to ensure that the BESS behaves in a realistic manner in the microgrid. All simulations are conducted for a single-day operation, representative of typical daily performance, capturing peak load and solar generation profiles.

## Improved bald eagle search algorithm for PV–BESS optimization

**Fig. 6:**
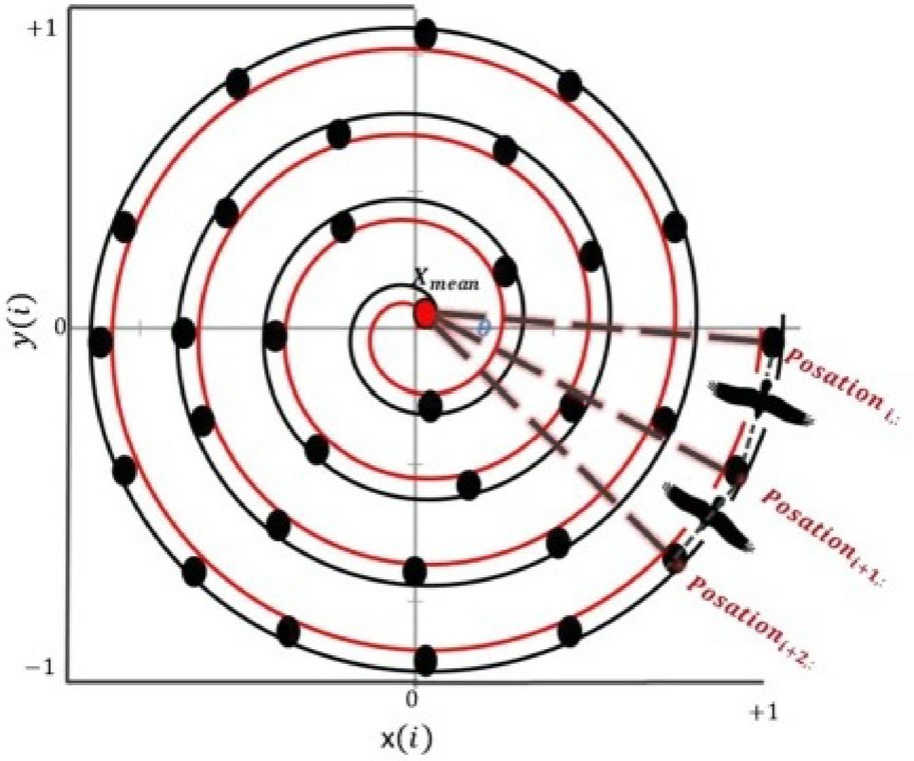
Bald eagle search phase with spiral movements^36^.

The Bald Eagle Search (BES) approach has been developed based upon a search strategy of American bald eagles that emphasizes their proficiency in opportunistic behaviors and their astounding accuracy in capturing prey. The BES algorithm follows eagles’ initial decision to explore a hunting opportunity and their propensity to abandon the search area if it is too energy demanding to search by limiting the search area to approximately 700 meters from its nest, or home base. The algorithm includes three major phases of hunting: one for area selection, one for searching, and one for diving. In the area selection phase, for example, bald eagles observe an area with possible further hunting opportunities marked by a number of prey items which is defined mathematically relative to a relationship that includes a parameter ‘a’, between 1.5 and 2, and a random number ‘rand’ that is between zero and one. In the search phase, eagles traverse the search area in a spiral movement while they hunt for prey and determine the best position to dive for hunting. This movement takes place in a polar scheme that incorporates the current, next, and central positions and adjusts movements in the vertical and horizontal direction. In the end, all positions in the polar scheme are between + 1 and − 1^34,35^. Bald Eagle Search Phase; spiral movement can be seen in Fig. 6.

21 $$\:{P}_{i,new}={P}_{i}+y\left(i\right)*\left({P}_{i}-{P}_{i+1}\right)+\mathrm{x}\left(\mathrm{i}\right)\mathrm{*}\left({P}_{i}-{P}_{mean}\right)$$

22 $$\:x\left(i\right)=\frac{xr\left(i\right)}{max\left(\left|xr\right|\right)}$$

23 $$\:y\left(i\right)=\frac{yr\left(i\right)}{max\left(\left|yr\right|\right)}\:\:\:\:$$

24 $$\:xr\left(i\right)=r\left(i\right)*\mathrm{sin}\left(\theta\:\left(i\right)\right),\:yr\left(i\right)=r\left(i\right)*\mathrm{cos}\left(\theta\:\left(i\right)\right)\:$$

25 $$\:\theta\:\left(i\right)=\:\mathrm{a}\:*\pi\:*rand$$

26 $$\:r\left(i\right)=\theta\:\left(i\right)+R*rand$$

**Fig. 7:**
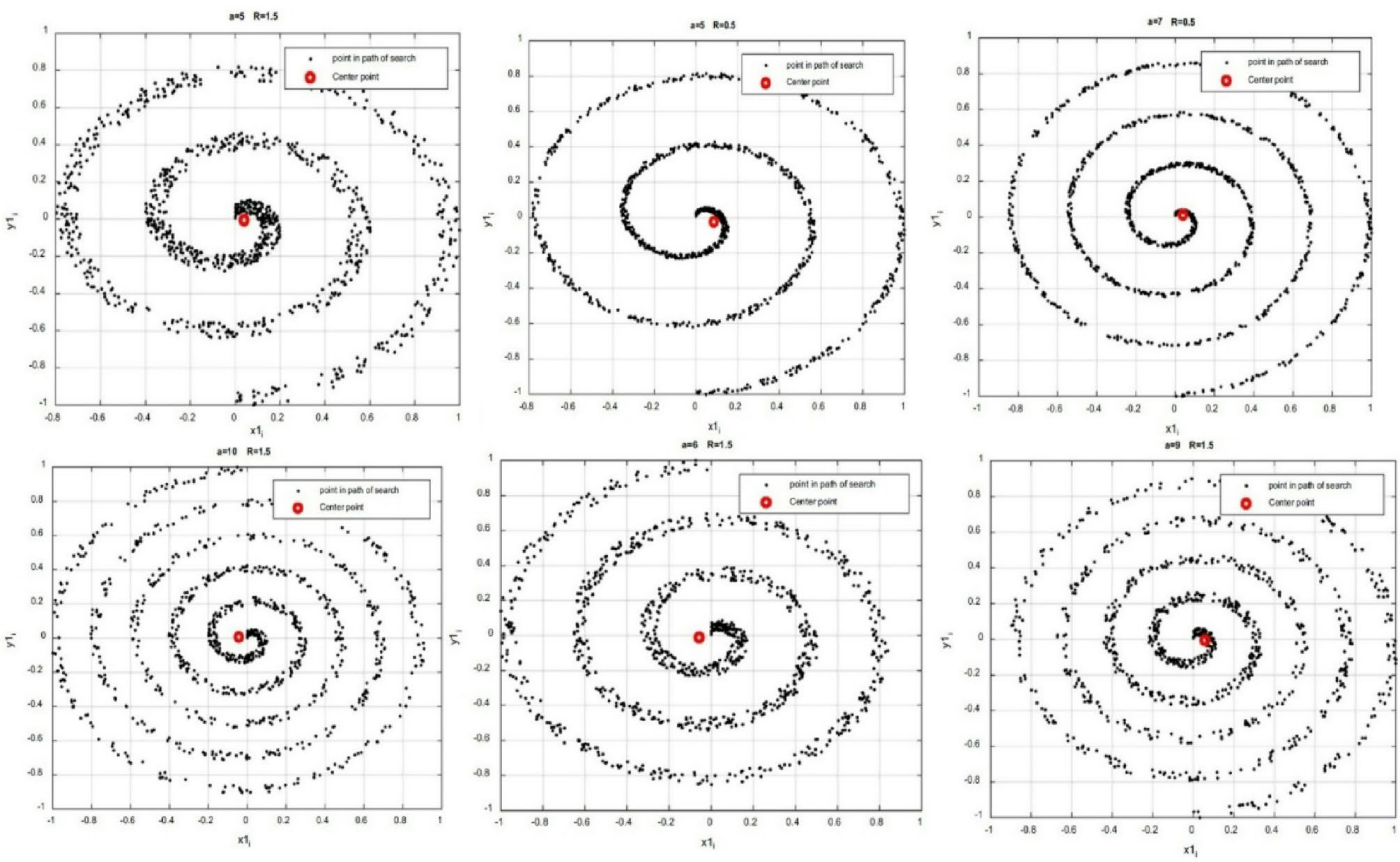
Changing the parameters *a* and *R* during the search^36^.

The parameter *a*, which is between 5 and 10, is used to determine the angle between the search point and the central point. The parameter *R* is used to determine the number of search cycles, which takes a value between 0.5 and 2 (Fig. 7).

The BES algorithm, which stands for Bald Eagle Search, is based on the way American bald eagles hunt for prey. This algorithm accurately depicts the eagle’s opportunistic behavior, strong precision, and impressive skill-set in capturing prey. For example, the bald eagle decides where to hunt based on a search radius of 700 meters around their nest since they are unable to wander too far from their nest due to energy constraints. The eagles carry out their hunting behavior in phases: select an area, search deeply for animals, and dive to hunt. In the selection phase, only the areas with a number of prey are selected by eagles and the area is represented by a mathematical function that has a biologically relevant, random variable ‘a’ that is between 1.5 and 2, and alongside a random number between zero and one, ‘rand’. In the search phase, while moving inside the selected search area, the eagle assumes a spiral motion to search for prey while determining in which position and orientation to dive while hunting^37^. The movement employs a polar framework, where the adjustments are made for the current point, the next point, and the center point, both vertically and horizontally. Then, all points in a polar framework will be confined to between + 1 and − 1. In the dive phase, the eagles circle above their target, then dive from the best position in the search space. The movement is again modeled around a polar equation framework, where the current point, center point, and best point are all adjusted vertically and horizontally. Parameters *C*_*1*_ and *C*_*2*_ define the strength of search movement of the bald eagle. The best solution is scaled by a random number and is used to guide it back towards the best central points. The flow chart of the planned method employed to identify and estimate the capacity of the photovoltaic resources within the distribution feeder is depicted in^38,39^.

The bald eagle algorithm runs the following process to locate and determine the optimal capacity of photovoltaic resources in the distribution system with the goals of reducing losses and improving the voltage profile.

- The first step is to enter the parameters related to the network under study, such as bus information, load amount, etc.
- The second step is to enter the parameters of the bald eagle algorithm, such as the number of eagles, the number of iterations, etc.
- The third step is that each eagle randomly selects a location that indicates the location of the installation of distributed photovoltaic generation resources with the appropriate capacity, load flow is taken and the value of the objective function is calculated.
- The fourth step is that when the best answer is obtained in the selection stage, the bald eagle spirals in search of prey and chooses the best position to dive. The values of each of the particles are placed in the system.
- The fifth step is that the best location identified by the bald eagles after performing the dive steps load flow is taken and the value of the objective function is calculated and the new obtained particles are sorted based on merit.
- The sixth step is if the flowchart condition is established, the work is finished. However, if the termination conditions are not met, the movement descends to step four and the bald eagle repeats its spiral movements to search for prey to get the best position to dive, and after that, the new particles are updated again and replace the old particles. That is, each eagle randomly selects a location for installing distributed generation resources with a new appropriate capacity.

In summary, a meta-heuristic optimization model of bald eagle search inspired by the nature of the hierarchical energy is created, and a multi-microgrid operation strategy that combines battery and power interaction to reduce operating costs. This study performs dynamic optimization to achieve the optimal economic benefits of the active distribution network with a multi-microgrid system. An improved bald eagle search algorithm has been developed to achieve the optimal energy configuration by determining the optimal, cross, and competitive capacity and location. The superiority of the bald eagle search algorithm has been shown by the results of various test functions and evaluation indicators^16,40^.

**Fig. 8:**
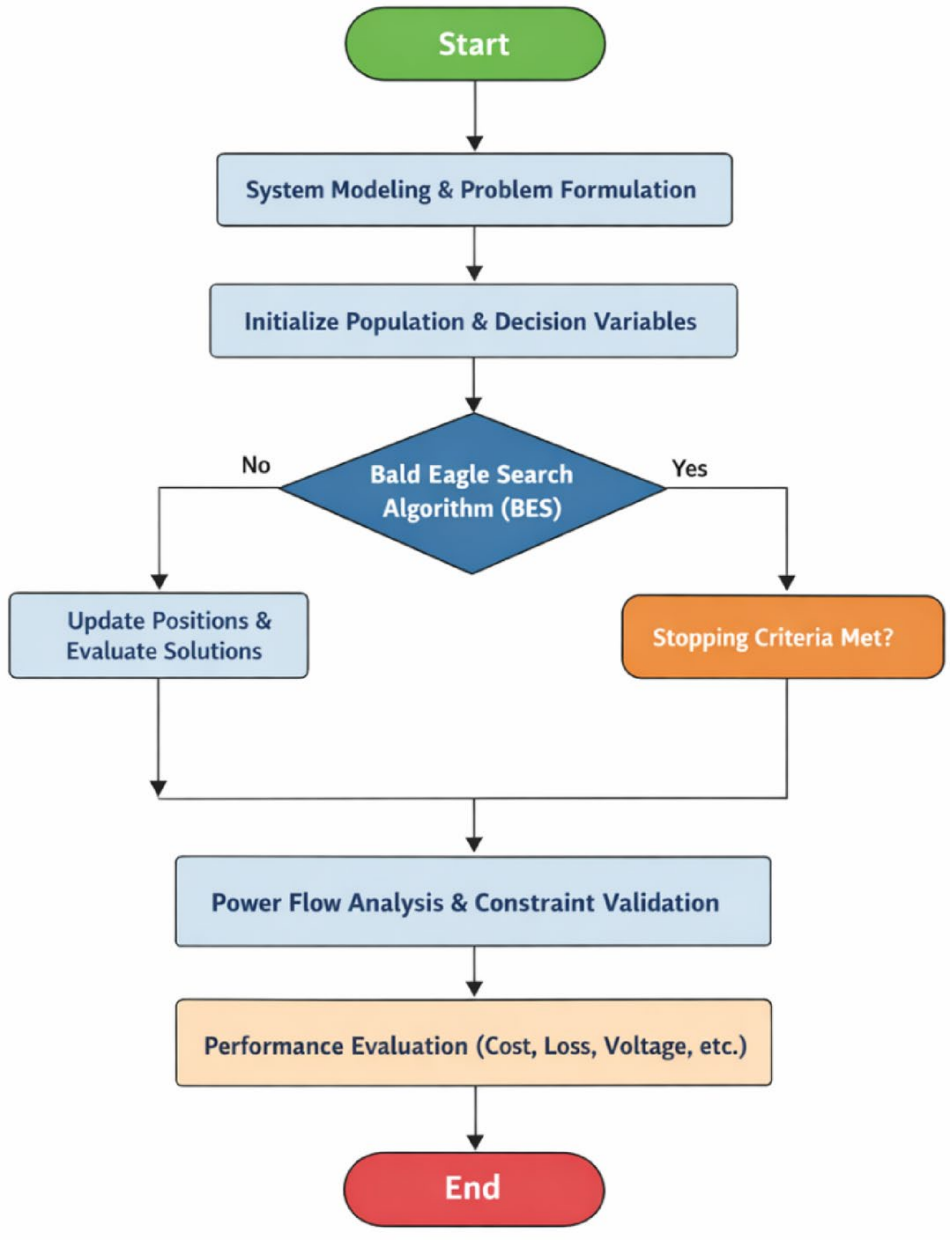
Flowchart of the proposed optimization framework.

Figure 8 presents the flowchart of the overall optimization process. The optimization process begins with system modeling and problem formulation, and then the initialization of decision variables. The Bald Eagle Search algorithm is then employed to optimize the sizing and placement of PV and BESS units, and finally, the optimal solutions are analyzed using power flow analysis.

In this study, the standard Bald Eagle Search (BES) algorithm has been improved to enhance the convergence speed, solution quality, and robustness of PV and BESS allocation. The key improvements include:

1. Adaptive Parameter Control: Parameters that control exploration and exploitation are adjusted adaptively throughout the iterations to achieve a balance between global and local search.
2. Lévy-flight Perturbation: A Lévy-flight perturbation method is incorporated to enable the search algorithm to move out of local optima and search the space more efficiently.
3. Constraint Handling Mechanism: A penalty function method is used to ensure that the network operation constraints (voltage limits, line currents, and power balance) are met.
4. Elitism Strategy: The best solutions obtained at the end of each iteration are retained to enhance convergence.

In the improved BES algorithm, control parameters are dynamically adjusted to balance exploration and exploitation. The adaptive update rule is defined as^39,40^:

27 $$\:\theta\:\left(t\right)={\theta\:}_{min}+({\theta\:}_{max}-{\theta\:}_{min})(1-\frac{t}{T})$$

where $\:\theta\:\left(t\right)$ represents a control parameter such as ω, µ, or λ, t is the current iteration, and T is the maximum number of iterations. θ_max_ and θ_min_ define the upper and lower bounds of the parameter, respectively. To improve global search capability and avoid local optima, Lévy flight is used in the exploration phase^39,40^:

28 $$\:{X}_{t+1}={X}_{t}+\alpha\:\:\mathrm{L}\acute{e}\mathrm{v}\mathrm{y}\left(\beta\:\right)$$

where X_t_ is the current solution, α is the step size scaling factor, and β is the Lévy distribution parameter controlling step length. The Lévy distribution is defined as^39,40^:

29 $$\:\mathrm{L}\acute{e}\mathrm{v}\mathrm{y}\left(\beta\:\right)\:\sim\:u=\frac{1}{{\left|v\right|}^{1/\beta\:}}$$

where µ and ν are random variables drawn from a normal distribution.

The proposed improvements are used to enhance exploration–exploitation balance (adaptive control), avoid local optima (Lévy flight), and improve convergence stability (elitism), which are essential for solving the complex PV–BESS optimization problem.

A comparison between the standard Bald Eagle Search (BES) algorithm and the improved version used in this study is provided to highlight the modifications introduced in the proposed method (Table 3). The improved BES incorporates adaptive parameter control, Lévy flight exploration, elitism, and explicit constraint handling, which are not present in the standard formulation. These enhancements lead to improved convergence stability and better solution quality in the PV–BESS optimization problem.

**Table 3:** Comparison of Standard BES and Improved BES.

Feature	Standard BES	Improved BES (This Work)
Exploration	Basic search mechanism	Lévy flight-based exploration
Parameter control	Fixed parameters	Adaptive parameters
Elitism	Not included	Included
Constraint handling	Implicit	Explicit
Convergence behavior	Moderate	Improved and stable
Optimization performance	Standard	Enhanced (lower cost/loss)

These modifications result in faster convergence, lower energy losses, and better voltage profiles than the standard BES algorithm. The enhanced BES algorithm is implemented using the pseudocode presented in Algorithm 1. It integrates adaptive parameter control, Lévy flight-based exploration, exploitation toward the best solution, elitism preservation, and penalty-based constraint handling to ensure feasibility and improve convergence performance.

**Figure:**
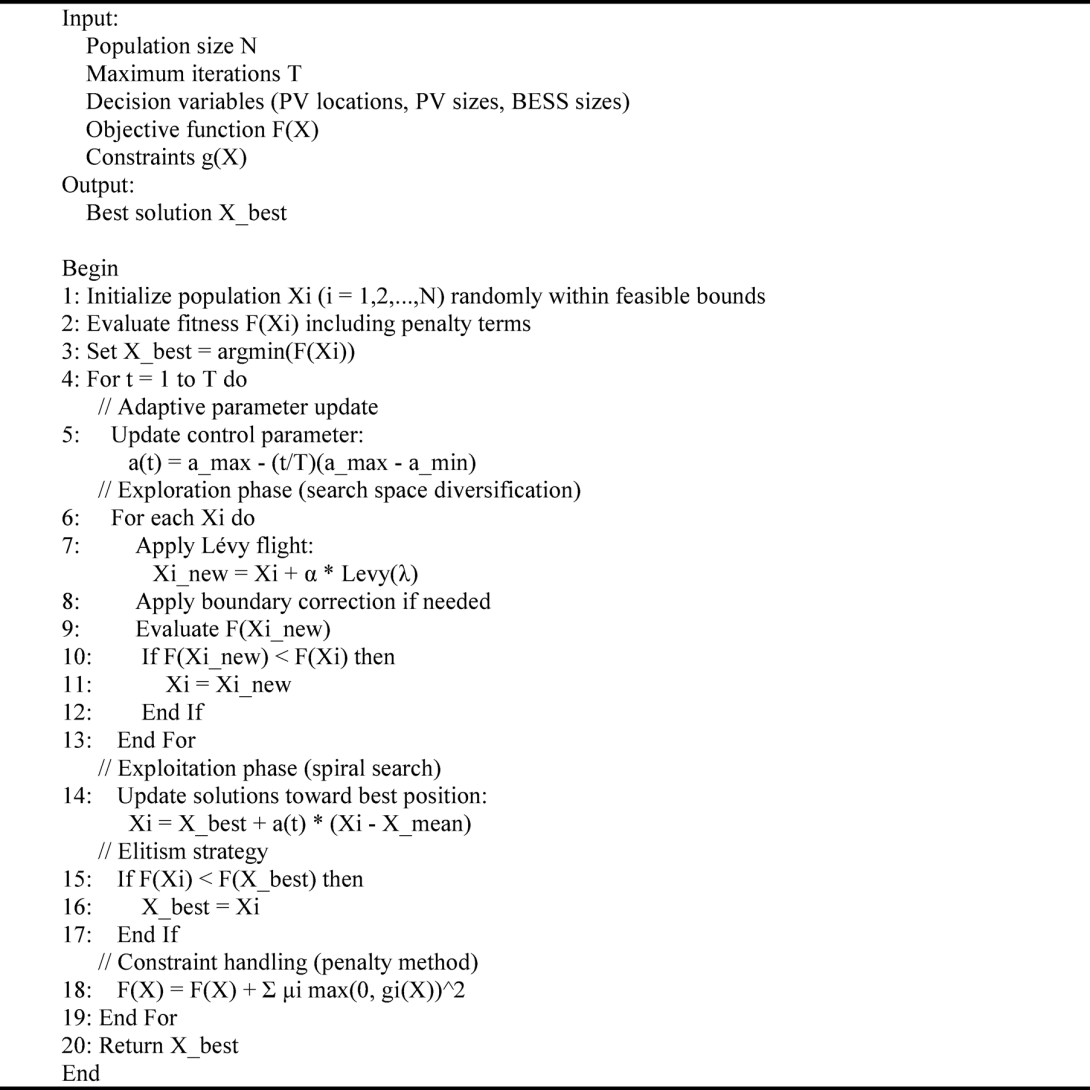
**Algorithm 1**: Enhanced Bald Eagle Search (EBES) Algorithm.

## Simulation results and discussion

One of the major challenges of specialists and operators of electrical power distribution networks is optimizing the daily operating costs of energy distribution. Underlying factors increase the expenses of a power distribution system, particularly those that incur plant losses, as a key determinant to overall costs. Similarly, specialists have noted that deploying distributed generation technologies, especially photovoltaics, has been shown to reduce losses thereby lowering operating costs and improving voltage dynamics of operating network system. This is the intended focus for the chapter of this paper, where we analyze and explore the outcomes of simulation experimentation in the MATLAB software application. In this section, the efficacy of the proposed optimization framework is assessed through simulation studies carried out on the test system. Two scenarios are considered:

- Scenario 1, including only PV integration (PV only),
- Scenario 2, including both PV and BESS integration (PV + BESS only).

The results are analyzed and compared with the benchmark algorithms based on their economic viability, reduction in power loss, and improvement in voltage profile. Due to the stochastic nature of the optimization algorithms, each algorithm is run 30 times independently for each test scenario. The 24-hour network loss is presented as the total daily loss to illustrate the overall reduction achieved by the integration of PV and BESS systems. The results presented are based on the mean, best, worst, and standard deviation values of the objective function and key performance indicators to ensure a statistically valid comparison. For all optimal solutions reported, the feasibility with respect to the physical system is checked for satisfaction of the network operational constraints. In particular, the bus voltages are within their allowed limits, the line currents are below their thermal limits, and the active and reactive power balance equations are met for all cases. The lower voltage limit is 0.95 pu; minor deviations at some buses after PV and BESS installation remain within acceptable operational limits. In both Scenarios, besides the Bald Eagle Search algorithm as the preferred choice, Genetic and Whale algorithms were also employed. The algorithms’ different features are described in Table 4.

**Table 4:** Optimization algorithm coefficients.

GA	Population	Iteration	P_m_	P_c_
	30	50	0.65	0.35
WOA	Population	Iteration	ω	υ
	30	50	0.06	0.76
BES	Population	Iteration	λ	µ
	30	50	0.93	0.03

The proposed BES, GA, and WOA algorithms are employed to optimize the placement and sizing of PV and BESS in the examined 69-bus feeder. Prior to the presentation of the percentage reduction in operating costs and power losses, the reference case (base case) without the integration of PV and BESS is established. In the base case, the total operating cost is 8.31 × 10⁷ $/year, the daily energy losses are 6430 kWh/day, and the minimum bus voltage is 0.930 p.u. for Scenario 1 and Scenario 2.

### Scenario 1: PV-only integration case

In the first Scenario, the localization and capacity of photovoltaic generation sources for the distribution feeder in study, without battery storage, were studied using the proposed Bald Eagle Search (BES) algorithm with the Genetic Algorithm (GA) and Whale Optimization Algorithm (WOA). In some projects, the batteries are eliminated from the system to save on initial costs and in Scenario 1; we will quantify the value of not using a battery in the photovoltaic generation system.

**Fig. 9:**
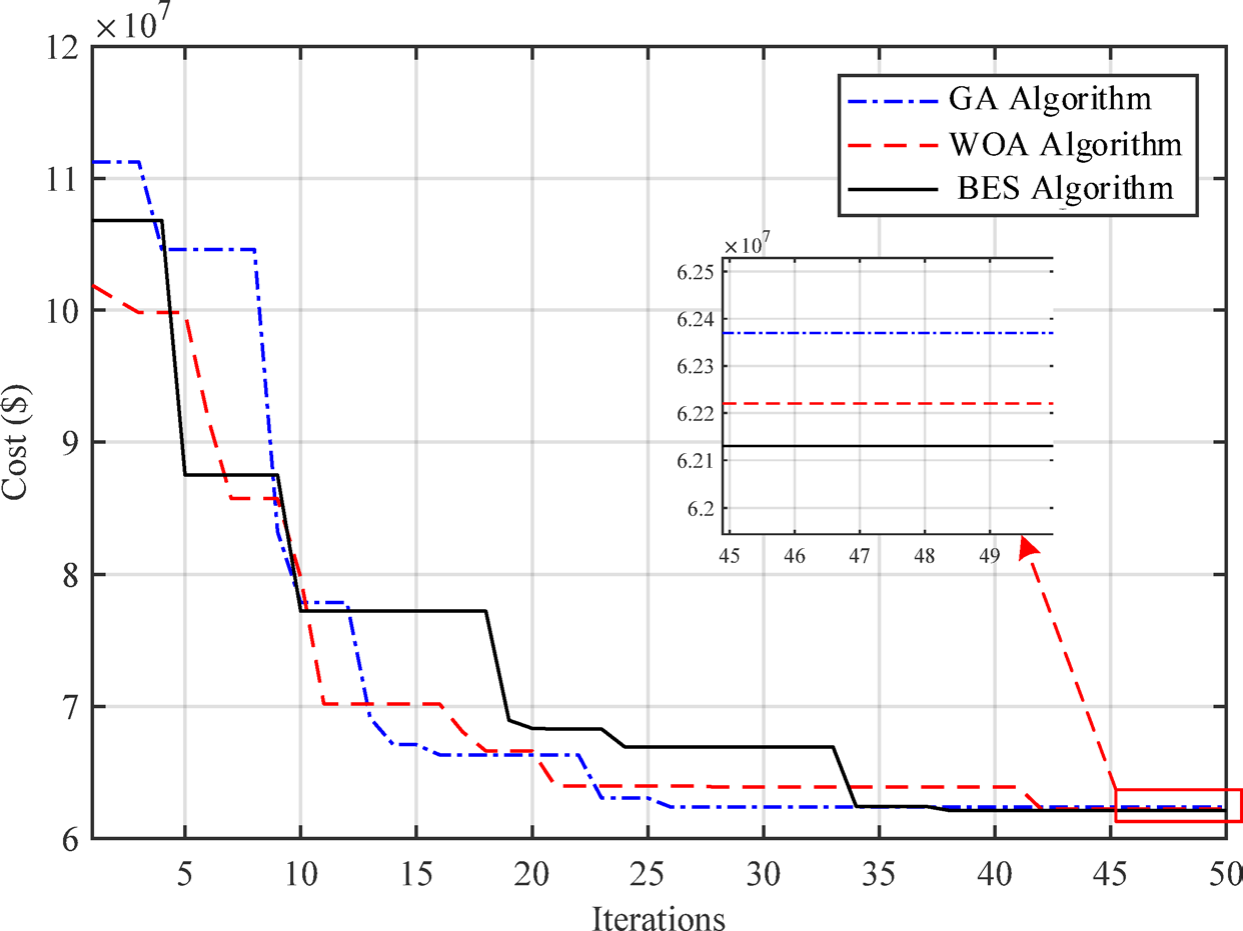
Convergence of Optimization Algorithms in Scenario 1.

The convergence curves in Fig. 9 illustrate the performance of the optimization algorithms in determining optimal locations for photovoltaic (PV) generation units. As observed, the Bald Eagle Search (BES) algorithm converges to a final objective value of 6.184 × 10⁷, compared to 6.241 × 10⁷ for the Genetic Algorithm (GA) and 6.219 × 10⁷ for the Whale Optimization Algorithm (WOA), indicating superior performance of BES.

Accordingly, the operating cost is reduced by 25.63% for the BES-based solution. The improved performance of all algorithms is further summarized in Table 5, which reports the optimal PV placement (PV1–PV8 in kW) for each method (BES, WOA, and GA), along with the corresponding minimum voltage levels (p.u.).

**Table 5:** Optimization Results in Scenario 1.

	PV1	PV2	PV3	PV4	PV5	PV6	PV7	PV8
BES Algorithm								
Installation Location	4	25	30	44	47	52	56	61
PV Capacity (kW)	31	118	86	243	84	218	204	207
Minimum Voltage (p.u.)	0.943							
WOA Algorithm								
Installation Location	4	28	35	40	47	49	58	64
PV Capacity (kW)	28	119	88	192	76	251	227	231
Minimum Voltage (p.u.)	0.935							
GA Algorithm								
Installation Location	4	24	34	38	49	51	60	63
PV Capacity (kW)	32	111	84	239	78	197	204	240
Minimum Voltage (p.u.)	0.941							

The Bald Eagle Search (BES) algorithm allocates PV units at nodes 4, 25, 30, 44, 47, 52, 56, and 61 with capacities of 31, 118, 86, 243, 84, 218, 204, and 207 kW, respectively, increasing the minimum network voltage to 0.943 p.u. In comparison, the Whale Optimization Algorithm (WOA) places PV units at nodes 4, 28, 35, 40, 47, 49, 58, and 64 with capacities of 28, 119, 88, 192, 76, 251, 227, and 231 kW, respectively, resulting in a minimum voltage of 0.935 p.u. The Genetic Algorithm (GA) selects nodes 4, 24, 34, 38, 49, 51, 60, and 63 with PV capacities of 32, 111, 84, 239, 78, 197, 204, and 240 kW, respectively, achieving a minimum voltage of 0.941 p.u. for the PV-only case without BESS. The corresponding total daily energy losses are 3869 kWh (GA), 3832 kWh (WOA), and 3791 kWh (BES), indicating that BES achieves the lowest losses and consequently the lowest operating cost among the compared methods.

**Fig. 10:**
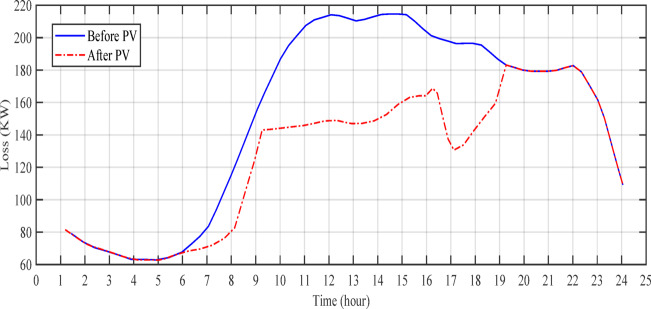
Power network losses before and after installation of photovoltaic sources.

The graphs in Fig. 10 illustrate the amount of energy loss in 24 h for the system designed with the Bald Eagle Search (BES) algorithm and the energy loss before the installation of photovoltaic sources.

The percentage reduction in losses after the implementation of installing photovoltaic generation sources in the studied feeder is calculated to be approximately 41%. With the help of using photovoltaic generation sources in distribution networks, in addition to measuring the reduction of ohmic losses and the reduction of operating costs, the network voltage profile will also be improved.

**Fig. 11:**
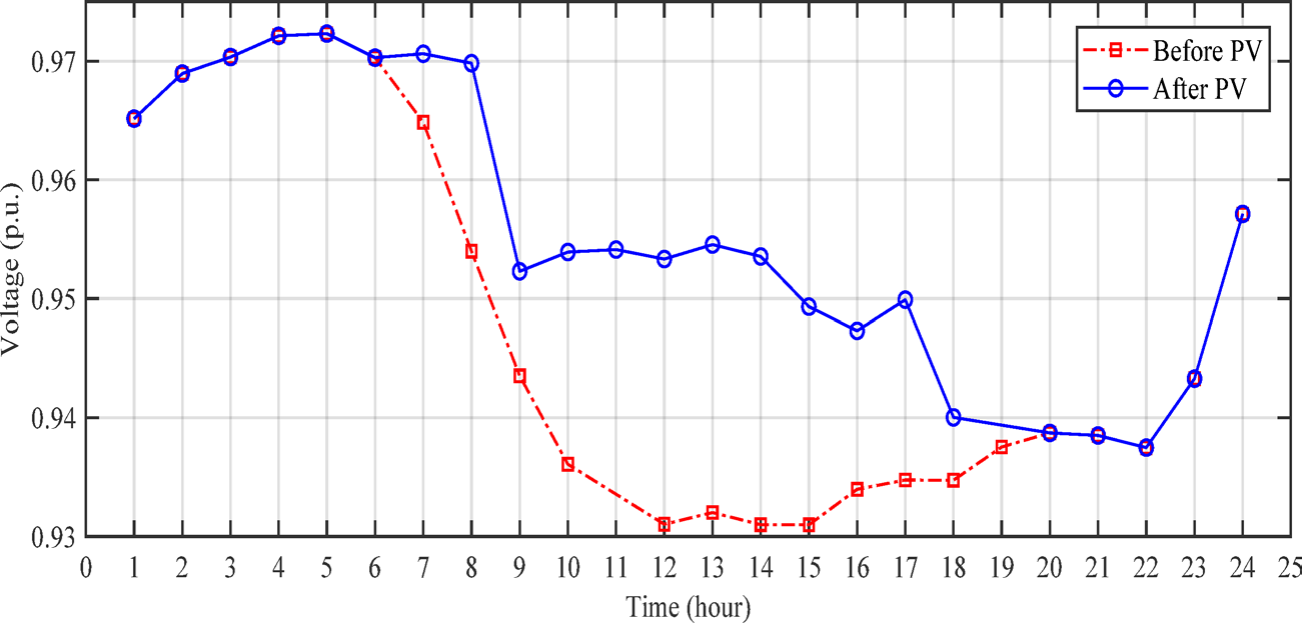
Power system voltage range before and after installation of photovoltaic sources.

The changes in the minimum network voltage value before and after the installation of photovoltaic generation sources are shown in Fig. 11. The most effective results are only shown from the proposed Bald Eagle Search (BES) algorithm, while the results of the other algorithms are removed from this figure, which is due to the greater efficiency and better performance of the BES algorithm compared to other methods. The positive effect of utilizing photovoltaic generation sources in improving power quality and increasing the minimum voltage range is evident by the voltage profile in the above diagram. Initially, the minimum voltage value in the studied feeder was approximately 0.93 per unit. With the implementation of installation and commissioning measures of photovoltaic arrays in the feeder, this minimum value has increased to about 0.941 per unit.

The results of the comparison between the different optimization algorithms for Scenario 1, where only PV sources are connected to the microgrid, are shown in Table 6. The table includes the total operating cost, daily power losses, percentage of loss reduction, minimum bus voltage, and standard deviation after 30 independent runs. As can be seen, the BES algorithm has the lowest cost and the highest percentage of loss reduction.

**Table 6:** Comparison of optimization algorithms in scenario 1.

Algorithm	Total Operating Cost ($/year)	Energy Loss (kWh/day)	Loss Reduction (%)	Min Voltage (*p*.u.)	Std (30 runs)
Base case	8.31 × 10⁷	6430	–	0.930	–
GA	6.24 × 10⁷	3869	39.8%	0.941	4.1 × 10⁵
WOA	6.22 × 10⁷	3832	40.4%	0.935	3.6 × 10⁵
BES	6.18 × 10⁷	3791	41.0%	0.943	2.1 × 10⁵

### Scenario 2: PV and BESS integrated case

In the second scenario of the simulations, the proposed Bald Eagle Search algorithm, along with the Genetic Algorithm and Whale Optimization Algorithm, were used to locate and allocate the capacity of photovoltaic and battery sources in the studied distribution feeder. The purpose of this scenario is to investigate the effect of battery participation on the operating costs of the distribution network. By adding batteries to the system, the initial costs will increase, but it needs to be investigated whether the reduction of losses can offset the costs of purchasing batteries.

**Fig. 12:**
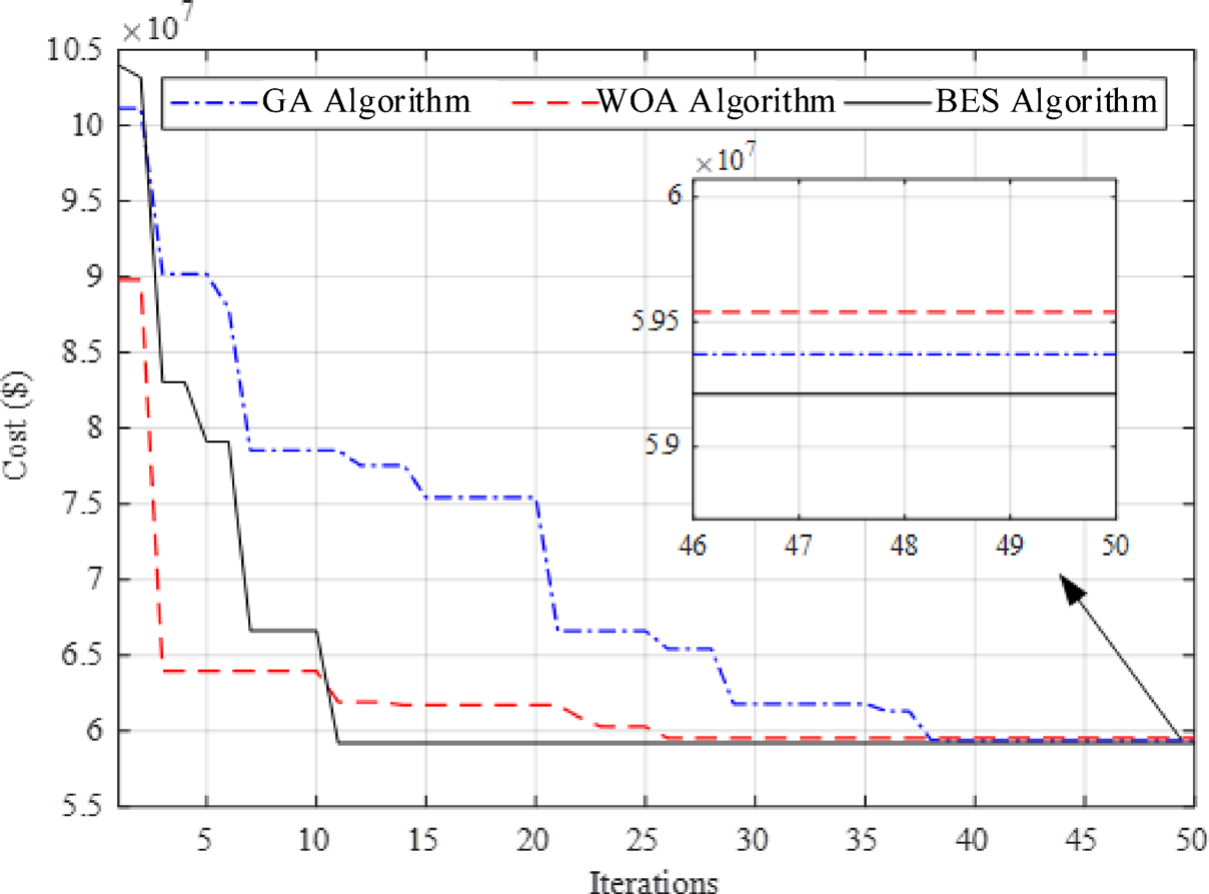
convergence of Optimization Algorithms in Scenario 2.

Figure 12 illustrates the convergence behavior of the optimization algorithms under the defined conditions of the second scenario. In the convergence curves of the optimization algorithms, the optimization results by these algorithms are detailed in Table 7, which shows the installation location, battery capacity (in kWh), photovoltaic capacity (in kW), and minimum voltage (in p.u.) for each algorithm (BES, WOA, and GA) at various PV locations (PV1 through PV8). Table 7 presents the optimal installation locations for both PV and BESS systems. The optimization algorithm may assign PV and BESS to different buses if this leads to improved network performance and cost efficiency.

**Table 7:** Optimization results in scenario 2.

	PV1	PV2	PV3	PV4	PV5	PV6	PV7	PV8
BES Algorithm								
Installation Location	5	26	32	48	51	53	62	64
Battery Capacity (kWh)	1190	443	1778	367	253	158	310	893
PV Capacity (kW)	25	81	80	179	66	170	178	188
Minimum Voltage (p.u.)	0.942							
WOA Algorithm								
Installation Location	5	24	32	41	49	50	62	64
Battery Capacity (kWh)	1328	433	1711	361	263	154	350	816
PV Capacity (kW)	25	83	80	164	59	185	190	187
Minimum Voltage (p.u.)	0.938							
GA Algorithm								
Installation Location	6	23	33	42	48	51	61	64
Battery Capacity (kWh)	1115	481	1923	336	260	162	362	931
PV Capacity (kW)	23	83	73	183	63	166	181	183
Minimum Voltage (p.u.)	0.940							

Under the proposed framework, the Bald Eagle Search (BES) algorithm selects nodes 5, 26, 32, 48, 51, 53, 62, and 64 for PV units rated at 25, 81, 80, 179, 66, 170, 178, and 188 kW, respectively, together with BESS capacities of 1190, 443, 1778, 367, 253, 158, 310, and 893 kWh, yielding a minimum voltage of 0.942 p.u. Similarly, the Whale Optimization Algorithm (WOA) allocates PV units of 25, 83, 80, 164, 59, 185, 190, and 187 kW at nodes 5, 24, 32, 41, 49, 50, 62, and 64, with corresponding BESS ratings of 1328, 433, 1711, 361, 263, 154, 350, and 816 kWh, improving the minimum voltage to 0.938 p.u. Finally, the Genetic Algorithm (GA) places PV units of 23, 83, 73, 183, 63, 166, 181, and 183 kW at nodes 6, 23, 33, 42, 48, 51, 61, and 64, along with BESS capacities of 1115, 481, 1923, 336, 260, 162, 362, and 931 kWh, resulting in a minimum voltage of 0.940 p.u.

In the hourly analysis, the effect of optimizing distributed renewable generation sources and the use of battery storage by the Genetic algorithm, the Whale algorithm, and finally the proposed Bald Eagle Search algorithm is estimated to be approximately 3565 kWh, 3501 kWh, and 3427 kWh, respectively. The lowest losses in the power system associated with photovoltaic sources and battery storage with capacity determined by the BES algorithm are located, which leads to a reduction in operating costs of the power system.

**Fig. 13:**
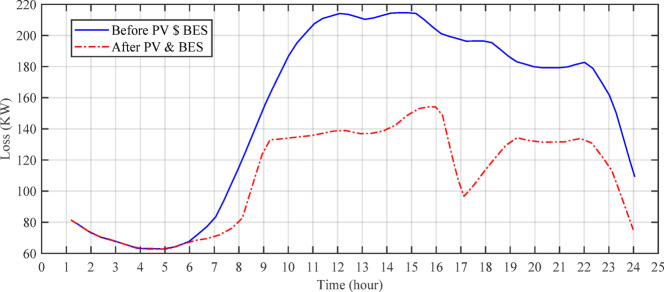
Power system losses before and after installation of photovoltaic and battery sources.

In Fig. 13, the line losses over 24 h for the system designed by the proposed BES algorithm and the losses before the installation of renewable sources and battery storage are depicted. it is observed from this image that the losses have decreased compared to the situation before the installation of renewable sources in the desired feeder. For example, at hour 12, the feeder losses were around 220 kW, but after the installation of photovoltaic generation sources and battery storage, this amount was reduced to less than 137 kW. Overall, the losses of this feeder have been reduced by about 55% compared to the situation before the modification.

The combination of photovoltaic generation sources with battery storage, in addition to reducing ohmic network losses and consequently reducing operating costs, also leads to improved voltage profile. The effect of improving the voltage profile also reasonably affects the reduction of losses.

**Fig. 14:**
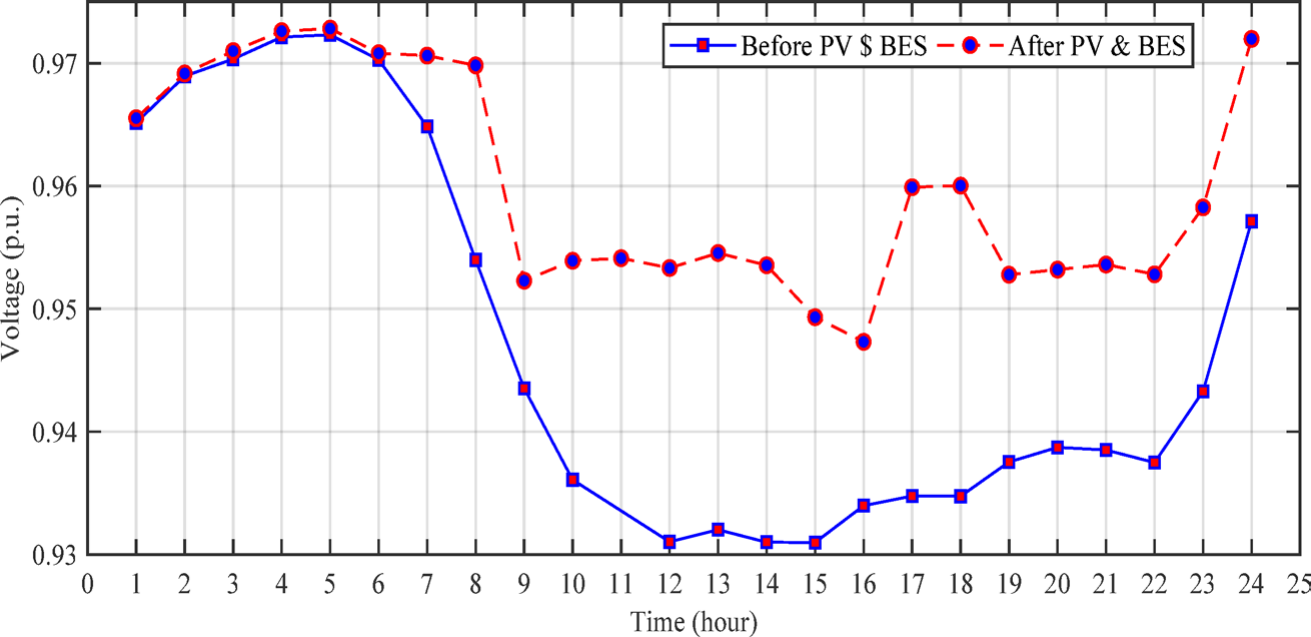
Power system voltage profile before and after installation of photovoltaic and battery sources.

**Fig. 15:**
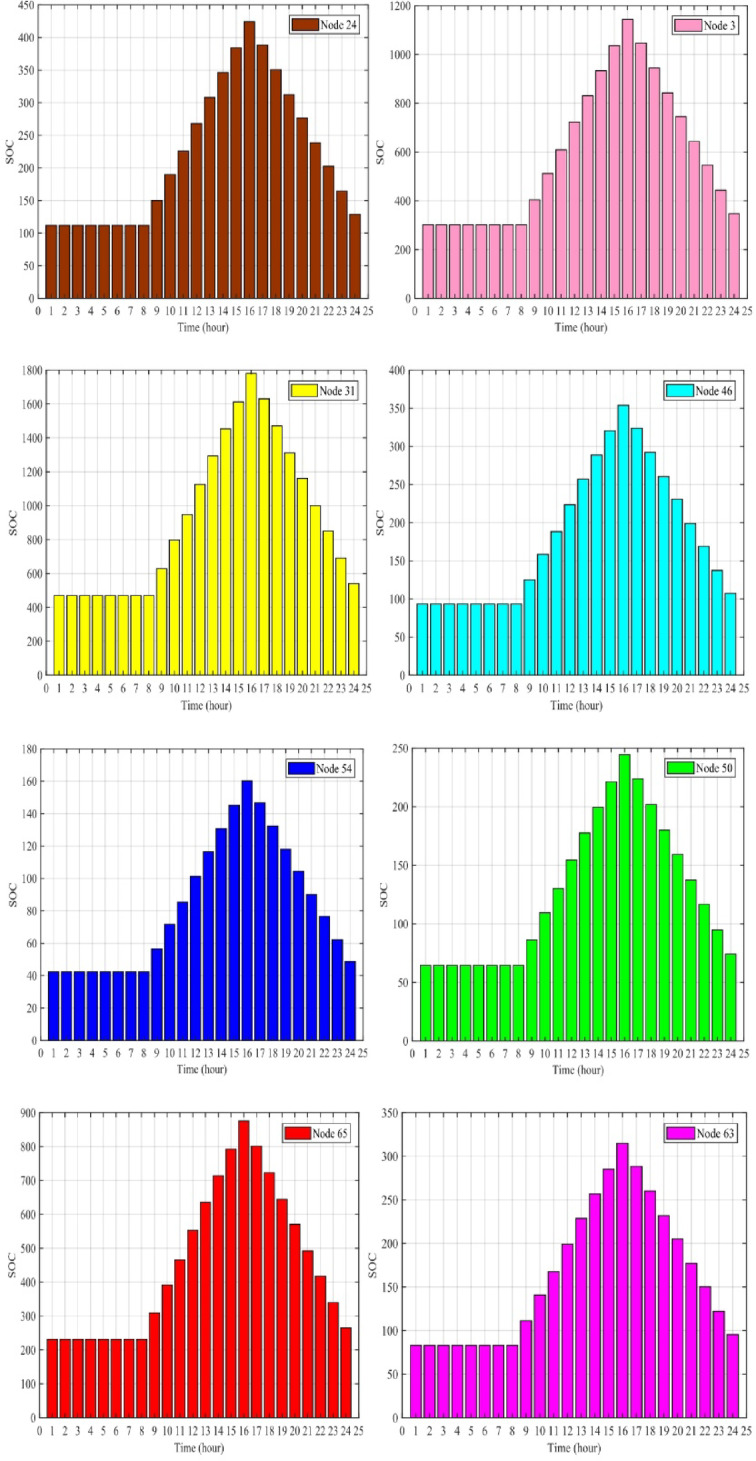
Battery state of charge.

In Fig. 14, the voltage profile of the studied power system, before the installation of photovoltaic and battery sources and after the implementation of optimization by the proposed BES algorithm, is depicted. The results of this figure have also proven the effect of using photovoltaic generation sources in improving the voltage profile and consequently improving power quality. The minimum voltage range in this feeder, before the installation of PV and battery arrays, has decreased to about 0.929 per unit, while after the installation of sources, the minimum voltage range has been calculated to be about 0.951 per unit. Subsequently, the state of charge (SOC) of the batteries during the 24-hour study is shown in Fig. 15. To prevent damage to the battery and increase its longevity, a minimum charge has been considered for the batteries so that the charge controller prevents the battery from discharging and does not reduce the amount of stored energy from a certain limit.

As is evident in the Fig. 15, the batteries are primarily charged in the hours before noon, and from 4:00 PM until they reach the minimum permissible charge level, they supply their energy to the grid. Utilizing batteries and storing excess energy from photovoltaic sources and providing it to the grid during hours when there is no solar radiation has been effective in reducing losses and operating costs.

The results for Scenario 2, where both PV and BESS systems are taken into account, are summarized in Table 8. The table contains the same set of performance metrics as in Table 6. The results show that the integration of BESS with PV systems further improves the cost and loss performance. The BES algorithm outperforms GA and WOA in terms of achieving a better trade-off between cost and voltage profile improvement while maintaining high solution consistency.

**Table 8:** Comparison of optimization algorithms in Scenario 2.

Algorithm	Total Operating Cost ($/year)	Energy Loss (kWh/day)	Loss Reduction (%)	Min Voltage (*p*.u.)	Std (30 runs)
Base case	8.31 × 10⁷	6430	–	0.93	–
GA	5.86 × 10⁷	3565	44.5%	0.940	3.9 × 10⁵
WOA	5.79 × 10⁷	3501	45.5%	0.938	3.2 × 10⁵
BES	5.77 × 10⁷	3427	55.0%	0.951	1.8 × 10⁵

### Performance validation and benchmark comparison

**Fig. 16:**
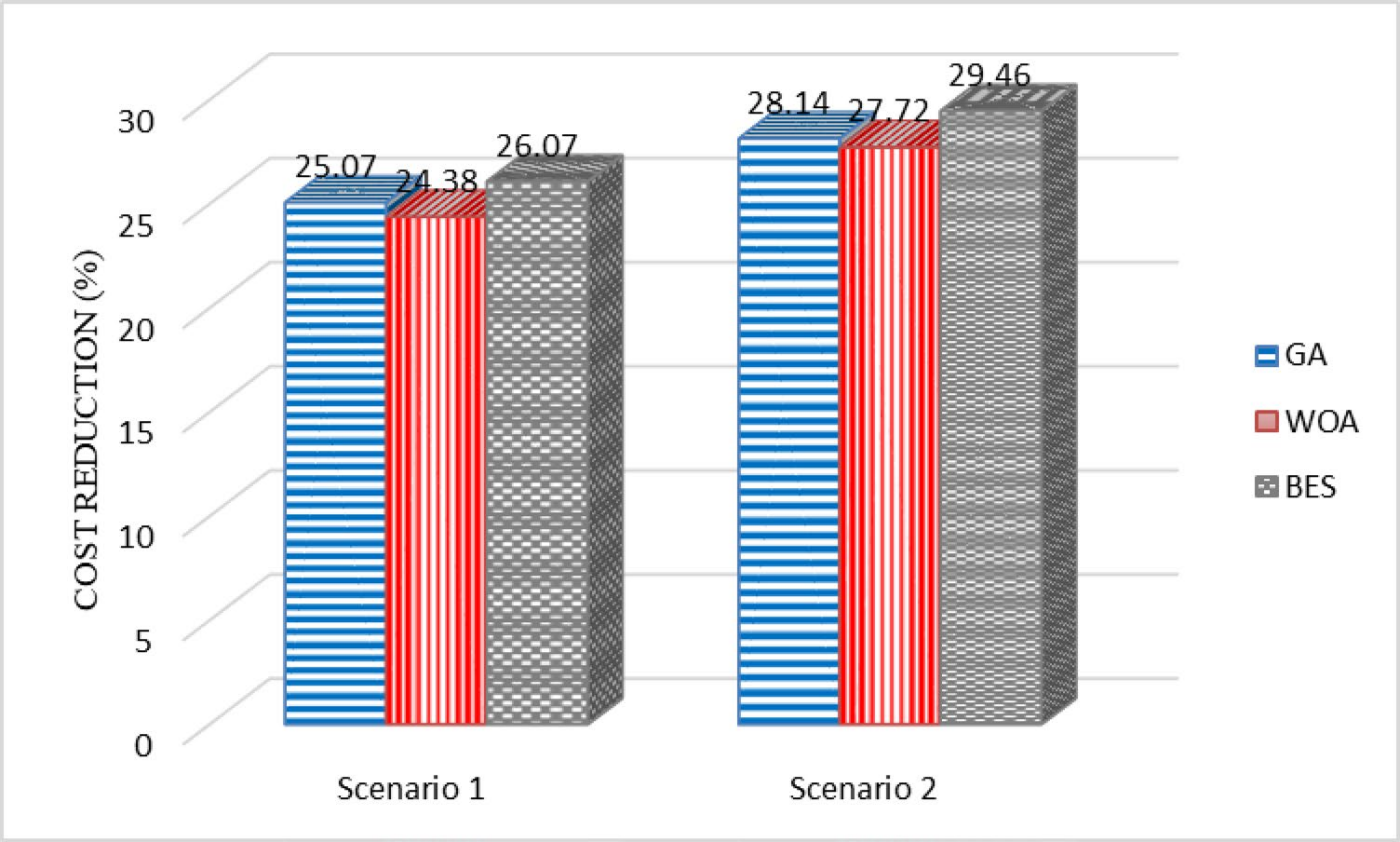
Percentage reduction of operating cost for GA, WOA, and BES under Scenario 1 and Scenario 2.

**Fig. 17:**
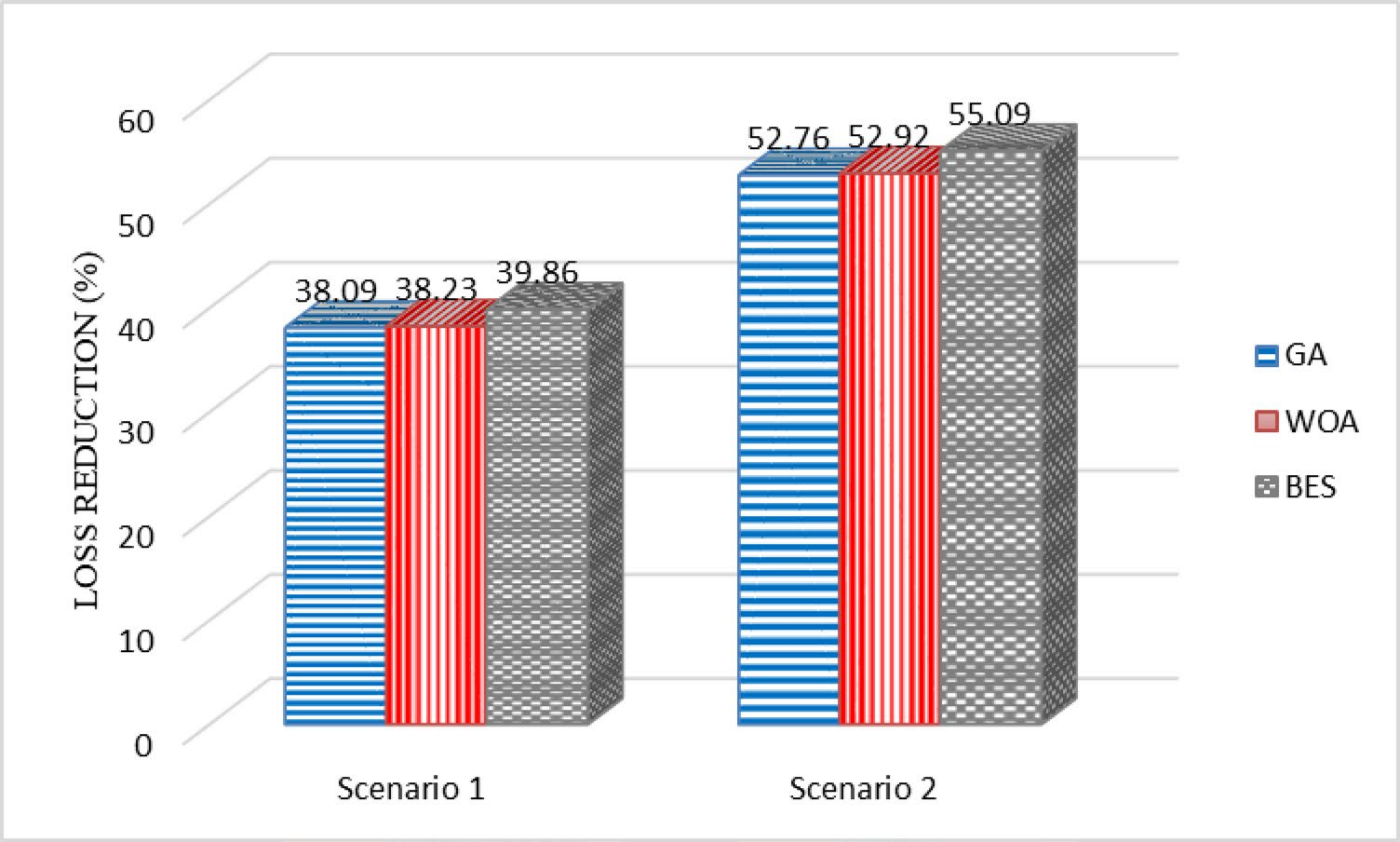
Percentage reduction of ohmic power losses for GA, WOA, and BES under Scenario 1 and Scenario 2.

**Fig. 18:**
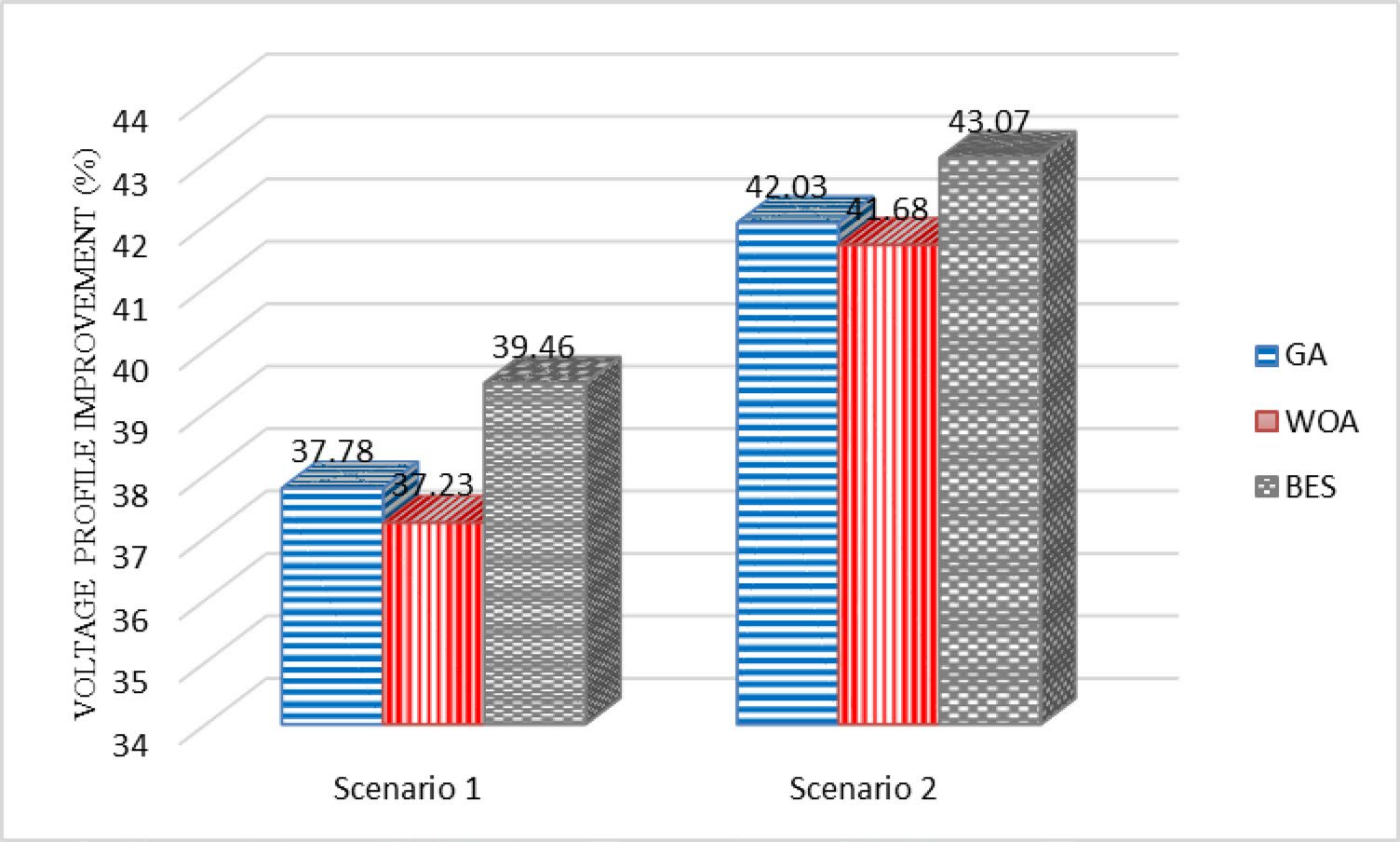
Percentage improvement of minimum voltage profile for GA, WOA, and BES under Scenario 1 and Scenario 2.

Subsequently, the optimization results obtained by the Genetic, Whale, and Bald Eagle Search optimization algorithms in the two defined scenarios were analyzed. To examine the results more closely, the percentage reduction in losses, the improvement in the voltage profile, and finally, the percentage reduction in operating costs over the studied time period were calculated and displayed as bar charts in Figs. 16, 17 and 18. Figures 16, 17 and 18 show the comparative performance analysis of the three optimization algorithms, namely Genetic Algorithm (GA), Whale Optimization Algorithm (WOA), and Bald Eagle Search (BES), for two different simulation scenarios. Scenario 1 represents the integration of photovoltaic (PV) systems only, whereas Scenario 2 represents the combined integration of PV and Battery Energy Storage Systems (BESS). Each of the figures shows the percentage improvement obtained by the three algorithms over the base case without any renewable energy and storage system installation.

As illustrated in the Fig. 16, the percentage reduction in operating costs achieved by the Bald Eagle Search (BES) algorithm in both scenarios is lower compared to the Genetic Algorithm (GA) and Whale Optimization Algorithm (WOA). In the case of photovoltaic (PV)-only integration, the operating cost is reduced by approximately 26.07%, whereas the integration of PV with battery energy storage systems (BESS) further decreases the operating cost to 29.46%.

The results of Fig. 17 confirm the very significant impact of the presence of the battery alongside the photovoltaic system in reducing network losses. Specifically, by using the battery in the system, the losses are reduced by 55%. By using the battery alongside the photovoltaic system, it is possible to have an effect in reducing voltage deviation in hours when there is no necessary solar radiation intensity. However, considering the load profile and load map throughout the day, the percentage improvement in the voltage profile in the two scenarios is relatively close to each other.

For the comprehensive validation of the proposed optimization framework, a comparative analysis has been performed on two benchmark distribution networks, namely the IEEE 69-bus and IEEE 33-bus systems. The IEEE 33-bus distribution system is a widely used benchmark radial network for evaluating distributed generation and optimization algorithms. All network data, including bus information, line parameters, and load specifications, are taken from^41^. Additionally, for both systems, three scenarios have been examined: the base case without any installation, PV-only integration, and integrated PV-BESS. As presented in Table 9, the results clearly show that the proposed method provides significant technical and economic enhancements for both test systems. For the IEEE 69-bus test system, the reduction of daily power losses by PV-only integration is around 41%, and further improvement in loss reduction to 55% is obtained by the integration of PV and BESS, along with the lowest cost and best voltage profile.

Similarly, for the IEEE 33-bus system, the reduction in loss for the PV-only system is 41.3%, whereas for the PV-BESS system, it is 52%, and the improvement in the minimum bus voltage from 0.941 p.u. to 0.962 p.u. is achieved. The above results clearly show that the proposed framework has a strong generalization capability and is not restricted to a particular network topology. Overall, this validation study clearly shows that the installation of PV systems is economically viable compared to the base case, and the addition of BESS further improves the system performance. Thus, the combination of PV and BESS systems offers the most effective and robust solution for the optimal functioning of active distribution networks. In addition, a comparative analysis between GA, WOA, and BES was performed under identical conditions for both IEEE 33-bus and IEEE 69-bus systems. As shown in Table 9, BES consistently outperforms the other algorithms in terms of cost reduction, power loss minimization, and voltage improvement across both test systems.

**Table 9:** Comparative results for IEEE 33-bus and IEEE 69-bus systems.

Test System	Algorithm	Configuration	Operating Cost ($/year)	Energy Loss (kWh/day)	Loss Reduction (%)	Min Voltage (*p*.u.)
IEEE 33-bus	Base case	–	3.92 × 10⁷	2023	–	0.941
	GA	PV only	3.05 × 10⁷	1215	39.9%	0.951
	WOA		3.01 × 10⁷	1189	41.1%	0.953
	BES		2.95 × 10⁷	1187	41.3%	0.955
	GA	PV + BESS	2.72 × 10⁷	1010	50.2%	0.960
	WOA		2.66 × 10⁷	990	51.5%	0.961
	BES		2.61 × 10⁷	972	52.0%	0.962
IEEE 69-bus	Base case	–	8.31 × 10⁷	6430	–	0.930
	GA	PV only	6.24 × 10⁷	3869	39.8%	0.941
	WOA		6.22 × 10⁷	3832	40.4%	0.935
	BES		6.18 × 10⁷	3791	41.0%	0.943
	GA	PV + BESS	5.86 × 10⁷	3565	44.5%	0.940
	WOA		5.79 × 10⁷	3501	45.5%	0.938
	BES		5.77 × 10⁷	3427	55.0%	0.951

## Conclusion

This paper presented an enhanced Bald Eagle Search (BES) optimization framework for optimal siting and sizing of distributed PV and BESS units in radial distribution systems. The proposed model aims to minimize operating cost and energy losses while enhancing voltage profile performance. The effectiveness of the approach was evaluated on IEEE 33-bus and IEEE 69-bus benchmark systems and benchmarked against GA and WOA under identical simulation settings. The numerical results demonstrate that BES consistently provides superior performance across both test systems and operating scenarios. Specifically, in the IEEE 69-bus system, BES achieves up to 55% reduction in energy losses and improves the minimum voltage to 0.951 p.u. In the IEEE 33-bus system, it achieves a 52% reduction in energy losses while increasing the minimum voltage to 0.962 p.u., along with the lowest operating cost compared to GA and WOA. Furthermore, BES exhibits improved convergence characteristics and solution stability across multiple runs. It is worth noting that the analysis is based on balanced radial networks with deterministic load and renewable generation profiles. These assumptions may limit direct applicability under highly uncertain or unbalanced real-world conditions. Future work will extend the proposed framework to stochastic environments, unbalanced three-phase systems, and multi-objective formulations to further enhance practical applicability and operational robustness.

## Data Availability

All data generated or analysed during this study are included in this published article.
